# Design, synthesis and evaluation of anticancer activity of novel 2-thioxoimidazolidin-4-one derivatives bearing pyrazole, triazole and benzoxazole moieties

**DOI:** 10.1186/s13065-018-0418-1

**Published:** 2018-05-09

**Authors:** Heba A. Elhady, Refat El-Sayed, Hamedah S. Al-nathali

**Affiliations:** 1 Department of Chemistry, Faculty of Applied Sciences, Umm Al-Qura University, P. O. Box 13401, Makkah, 21955 Saudi Arabia; 2 Department of Chemistry, Faculty of Science, Al-Azhar University (Girls Branch), P.O. box 11754, Youssef Abbas Str., Cairo, Egypt; 3 Department of Chemistry, Faculty of Science, Benha University, Banha, Egypt

**Keywords:** 2-Thiohydantoin, Benzoimidazole, Benzoxazole, Pyrazole, HEPG-2 cell line and MCF-7 cell line

## Abstract

A novel series of substituted 2-thiohydantoin incorporated with benzoimidazole, pyrazole, triazole and/or benzoxazole moieties has been synthesized using (*E*)-3-[1-(4-bromophenyl)ethylideneamino]-2-thioxoimidazolidin-4-one **1** as the key starting material. The key material **1** also, reacted with an acetic anhydride, aromatic aldehydes, secondary amines, formaldehyde and triethyl orthoformate to give the corresponding acetyl, chalcone, Mannich bases and ethoxymethylene derivatives, respectively. The structures of the novel compounds were confirmed by spectral data and elemental analysis. The cytotoxic activity of all synthesized compounds was assessed in vitro against human hepatocellular cancer cell line (HePG-2) and breast carcinoma cell line (MCF-7). The bioassay results revealed that compound **14** has the best activity against HePG-2 cell line (IC_50_ = 2.33 μg/mL), while compound **5** has the best activity against MCF-7 cell line (IC_50_ = 3.98 μg/mL).

**Electronic supplementary material:**

The online version of this article (10.1186/s13065-018-0418-1) contains supplementary material, which is available to authorized users.

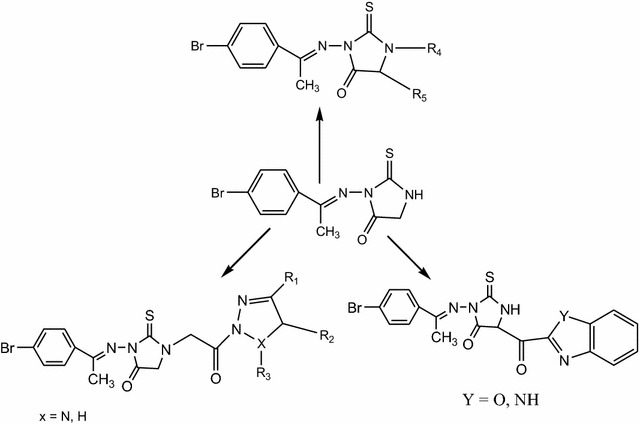

## Introduction

2-Thioxoimidazolidin-4-one ring (2-thiohydantoin) has been extensively studied. This five-membered heterocyclic ring is present in a wide range of biologically active compounds. The biological activities have been shown by some of their derivatives are mainly, anticonvulsant [1], antiviral [2], antiproliferative [3], anticancer [4–9], antibacterial, antifungal [10], anxiolytic [11], antidiabetic activity [12] and also used as inhibitor of a fatty acid amide hydrolase [13]. Additionally, 2-thiohydantoins are used in synthetic chemistry as in skin hyperpigmentation applications [14], in the production of antimicrobial polyurethane coatings [15], in textile printing, polymerization catalysis [16] and as a reagent for development of dyes [17]. The observed activities arise from the thiohydantoin heterocycle, but the different substituents attached to it are determinant in these properties. Diverse applications of 2-thioxoimidazolidin-4-one in drug field have encouraged the medicinal chemists to synthesize and evaluate a large number of novel molecules. In this research point, we design new compounds based on the biological activity of other heterocycles such as pyrazoles [18, 19], triazoles [20, 21], benzimidazole [22], benzoxazole [23] and Schiff bases [24–26] in the field of cancer and microbial therapy. As an extension of our work on the synthesis of heterocyclic systems and evaluation of their biological activity [27–33], we reported here the synthesis of some novel substituted 2-thiohydantoin and evaluate their cytotoxic activity. (*E*)-3-[1-(4-bromophenyl)ethylideneamino]-2-thioxoimidazolidin-4-one **1** was prepared and used as the building block for the synthesis of the novel compounds.

## Results and discussion

### Chemistry

**Scheme 1:**
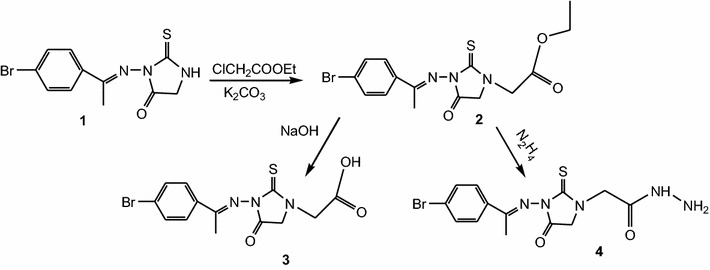
Synthesis of compounds **2**, **3** and **4**

**Scheme 2:**
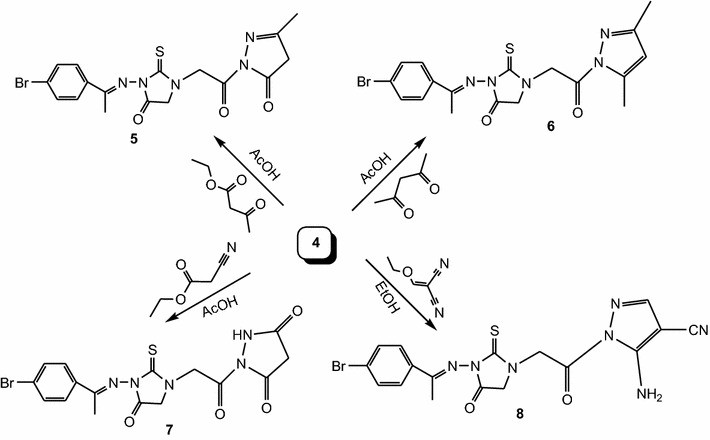
Synthesis of compounds **5**, **6**, **7** and **8**

As an extension of our interest on the chemistry of 2-thiohydantoin, we reported here the synthesis of novel derivatives using (*E*)-3-[1-(4-bromophenyl)ethylideneamino]-2-thioxoimidazolidin-4-one **1** as the key starting material. Compound **1** was prepared via reaction of (*E*)-2-[1-(4-bromophenyl)ethylidene]hydrazinecarbothioamide in the presence of sodium acetate [27, 28]. Alkylation of **1** with ethyl chloroacetate in the presence of anhydrous potassium carbonate gave (*E*)-ethyl 2-{3-[1-(4-bromophenyl)ethylideneamino]-4-oxo-2-thioxoimidazolidin-1-yl}acetate **2**. The structure of **2** was confirmed by spectral data, elemental analysis and chemical transformation. Thus, hydrolysis of the ester **2** with 2 N sodium hydroxide gave (*E*)-2-{3-[1-(4-bromophenyl)ethylideneamino]-4-oxo-2-thioxoimidazolidin-1-yl}acetic acid **3**. Hydrazinolysis of **2** with hydrazine hydrate in ethanol gave (*E*)-2-{3-[1-(4-bromophenyl)ethylideneamino]-4-oxo-2-thioxoimidazolidin-1-yl}acetohydrazide **4**, which is a suitable intermediate for the synthesis of the target compounds (Scheme 1). Cyclization of **4** with ethyl acetoacetate, acetylacetone and/or ethyl cyanoacetate in acetic acid gave the corresponding pyrazole derivatives **5**, **6** and pyrazole-3,5-dione derivative **7**, respectively. Also, reaction of **4** with ethoxymethylenemalononitrile (EMM) in ethanol under reflux gave pyrazole-4-carbonitrile derivative **8** (Scheme 2).

**Scheme 3:**
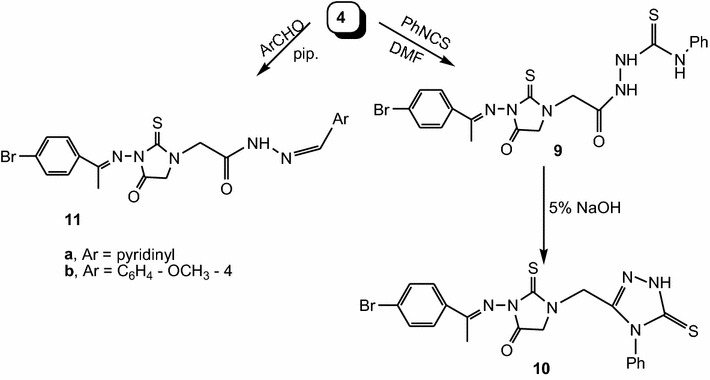
Synthesis of compounds **9**, **10** and **11 a**, **b**

To obtain a series of biologically active compounds, compound **4** was treated with phenylisothiocyanate in dimethylformamide to afford **9**, which cyclized with 5% alcoholic sodium hydroxide to give 4-phenyl-5-thioxo-1,2,4-triazole derivative **10**. Moreover, condensation of **4** with different aromatic aldehydes namely, isonicotinaldehyde and 4-hydroxy-3-methoxybenzaldehyde in ethanol in the presence of piperidine under reflux led to the formation of Schiff bases **11a**, **b** (Scheme 3).

**Scheme 4:**
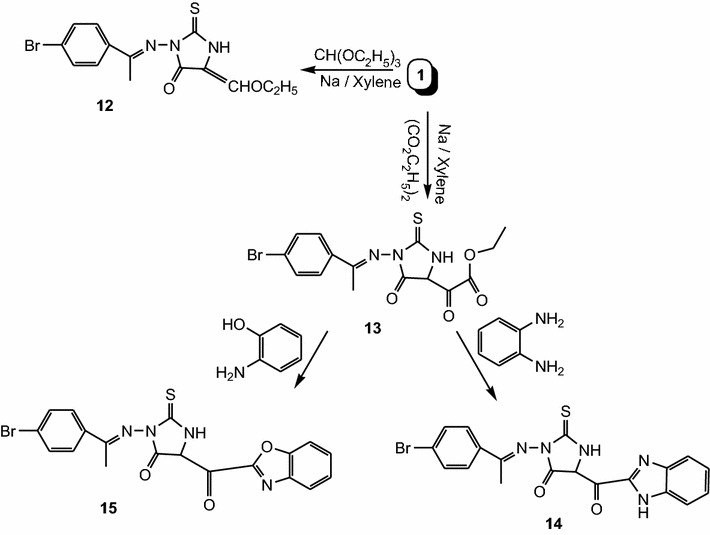
Synthesis of compounds **12**, **13**, **14** and **15**

**Scheme 5:**
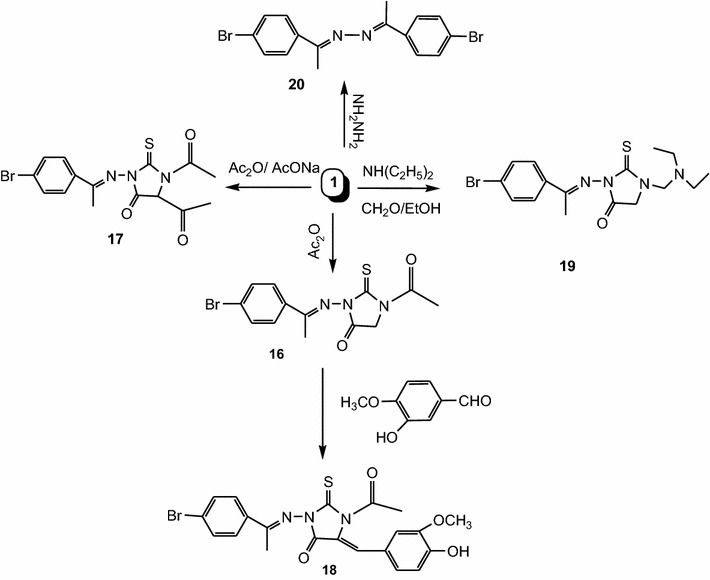
Synthesis of compounds **16**, **17**, **18**, **19** and **20**

To obtain substituted 2-thiohydantoin derivatives incorporated with benzoimidazole and/or benzoxazole moieties, compound **1** was reacted with triethyl orthoformate and/or diethyl oxalate in xylene in the presence of sodium metal under reflux to give **12** and/or **13**, respectively. Compound **13** was condensed with *o*-phenylenediamine and/or 2-aminophenol in acetic acid under fusion to give **14** and/or **15**, respectively (Scheme 4). Morover, new series of biologically active 2-thiohydantoin derivatives were prepared by acetylation of **1** with acetic anhydride to give **16** and **17**. Condensation of **16** with aldehydes such as vanillin in the presence of piperidine under fusion gave chalcone derivative **18**. Also, Mannich base was prepared by reacting **1** with diethylamine and formaldehyde in ethanol to give **19**. Finally, hydrazinolysis of **1** with hydrazine hydrate in ethanol gave **20** (Scheme 5). The structures of the synthesized compounds were confirmed by spectral data and elemental analysis.

### Biological assessment

#### In vitro anticancer screening

The anti-tumor activity of all synthesized compounds has been evaluated against two cell lines HepG-2 cells (human hepatocellular cancer cell line), and MCF-7 (breast carcinoma cell line) [34–36]. The cell lines were obtained from VACSERA Tissue Culture Unit, and the experiments were performed by the Regional Center for Mycology and Biotechnology, Al-Azhar University, Cairo, Egypt. Different concentrations of the tested samples (500, 250, 125, 62.5, 31.25, 15.6, 7.8, 3.9, 2 and 1 µg/mL) were used to detect the inhibitory activity. Cell viability (%) was determined by colorimetric method. Doxorubicin was used as the reference drug, as it is one of the most effective anticancer agents. The relationship between drug concentration and cell viability was plotted to obtain the survival curve of hepatocellular carcinoma cell line HePG2 and breast cancer cell line MCF-7. The IC_50_ value, which corresponds to the concentration required for 50% inhibition of cell viability was calculated.

Tables 1, 2, 3 show the in vitro cytotoxicity of the synthesized compounds against hepatocellular carcinoma cell line HePG-2. Tables 4, 5, 6 show the in vitro cytotoxicity against breast carcinoma cell line MCF-7. Data examination revealed that the tested compounds showed good to moderate activity. Compound **14** has the best activity against HePG-2 cell line (IC_50_ = 2.33 μg/mL), while, compound **5** has the best activity against MCF-7 cell line (IC_50_ = 3.98 μg/mL) and compound **11a** has the lowest activity against HePG-2 cell line (IC_50_ = 243 μg/mL) and against MCF-7 cell line (IC_50_ = 249 μg/mL). The structure and biological activity relationship of the title compound 1 showed that, the activity of thiohydantoin diverse with the substituents on it, where introducing active groups such as, CH_3_CO, OH, OCH_3_, OC_2_H_5_, =CH–OC_2_H_5_ enhanced the activity, also, the presence of benzoimidazole, pyrazolone, pyrazole carbonitrile and triazine moieties enhanced the activity of thiohydantoin, while the activity was decreased by introducing benzoxazole, pyrazolidinedione moieties and Schiff bases. Alkylation of thiohydantoin decreases the activity; however, when the ester **2** was reacted with hydrazine hydrate to form the acid hydrazide **4** the activity was enhanced especially against MCF-7 cell line.

**Table 1:** Cytotoxicity of compounds **1**, **2**, **3**, **4**, **5**, **6**, **7** and **8** against hepatocellular carcinoma cell line HePG2

Concentration µg/mL	Viability (%)/compound							
	**1**	**2**	**3**	**4**	**5**	**6**	**7**	**8**
**500**	5.26	19.86	2.97	7.48	3.04	31.67	12.58	6.31
**250**	10.68	28.74	7.28	14.53	6.31	46.23	20.64	14.96
**125**	18.97	39.45	11.49	21.84	10.25	68.92	31.75	20.85
**62.5**	25.14	68.17	16.85	29.41	16.43	83.64	42.31	26.43
**31.25**	34.72	79.52	23.72	38.63	22. 37	93.51	64.15	38.14
**15.6**	48.25	91.43	30.85	49.56	30.69	99.48	79.27	47.59
**7.8**	62.89	98.76	41.93	71.38	38.15	100	88.74	60.31
**3.9**	78.03	100	51.58	85.97	46.36	100	96.13	73.86
**2**	86.26	100	62.72	92.04	59.02	100	99.47	85.23
**1**	90.41	100	70.88	97.13	74.15	100	100	90.67
**0**	100	100	100	100	100	100	100	100

**Table 2:** Cytotoxicity of compounds **9**, **10**, **11a**, **11b**, **12**, **13**, and **14** against hepatocellular carcinoma cell line HePG2

Concentration µg/mL	Viability (%)/compound						
	**9**	**10**	**11a**	**11b**	**12**	**13**	**14**
**500**	8.43	8.32	23.84	10.32	4.29	4.83	3.74
**250**	15.82	16.01	48.67	19.47	11.82	12.56	8.91
**125**	24.67	23.65	72.89	27.93	20.49	19.74	14.82
**62.5**	30.93	29.86	90.31	38.76	26.54	28.63	20.94
**31.25**	41.28	37.40	98.16	50.37	32.75	35.16	25.86
**15.6**	48.71	46.89	100	68.24	41.87	46.29	31.43
**7.8**	62.39	58.62	100	81.49	55.46	57.18	37.82
**3.9**	78.24	73.94	100	90.65	73.82	70.42	45.27
**2**	88.65	81.47	100	97.34	81.46	83.29	50.94
**1**	94.27	89.53	100	100	88.73	89.64	62.35
**0**	100	100	100	100	100	100	100

**Table 3:** Cytotoxicity of compounds **15**, **16**, **17**, **18**, **19**, **20** and Doxorubicin against hepatocellular carcinoma cell line HePG2

Concentration µg/mL	Viability (%)/compound						
	**15**	**16**	**17**	**18**	**19**	**20**	Doxorubicin
**500**	6.49	4.31	6.78	4.68	3.84	6.91	3.6
**250**	11.25	11.72	15.93	10.35	9.65	11.76	3.97
**125**	19.43	18.25	24.85	17.46	13.81	20.95	6.43
**62.5**	30.61	23.87	34.91	25.72	19.46	32.47	9.68
**31.25**	39.45	30.64	42.87	32.69	25.83	44.35	16.45
**15.6**	51.26	38.92	67.29	44.82	32.17	76.97	23.87
**7.8**	67.18	47.30	83.97	51.96	39.68	89.50	30.69
**3.9**	84.91	64.59	91.48	64.53	48.54	94.16	37.54
**2**	92.73	76.28	98.02	72.31	61.83	98.73	42.91
**1**	96.48	81.49	100	79.48	69.42	100	48.76
**0**	100	100	100	100	100	100	100

**Table 4:** Cytotoxicity of compounds **1**, **2**, **3**, **4**, **5**, **6**, **7** and **8** against breast carcinoma cell line MCF-7

Concentration µg/mL	Viability (%)/compound							
	**1**	**2**	**3**	**4**	**5**	**6**	**7**	**8**
**500**	7.18	22.34	3.86	9.26	4.23	37.15	16.27	8.07
**250**	13.95	34.51	9.62	20.84	9.06	51.49	29.38	16.56
**125**	24.82	45.28	14.87	28.63	15.38	74.01	38.69	24.56
**62.5**	32.93	73.69	20.65	36.59	21.75	88.63	49.16	31.88
**31.25**	45.06	86.13	26.83	42.67	28.91	97.89	71.32	40.31
**15.6**	60.83	94.56	36.54	48.54	36.42	100	86.04	52.76
**7.8**	79.62	98.94	45.02	65.18	42.37	100	93.76	68.49
**3.9**	85.21	100	60.89	80.95	50.16	100	98.51	82.53
**2**	92.37	100	78.15	91.79	65.24	100	100	91.42
**1**	98.64	100	86.41	95.48	76.35	100	100	97.17
**0**	100	100	100	100	100	100	100	100

**Table 5:** Cytotoxicity of compounds **9**, **10**, **11a**, **11b**, **12**, **13**, **14** and **15** against breast carcinoma cell line MCF-7

Concentration µg/mL	Viability (%)/compound						
	**9**	**10**	**11a**	**11b**	**12**	**13**	**14**
**500**	8.09	6.48	31.79	8.51	6.37	5.96	5.08
**250**	17.24	15.72	49.81	20.38	14.75	11.74	10.24
**125**	26.85	24.16	78.26	32.75	23.81	20.82	18.76
**62.5**	37.51	35.29	89.47	45.06	34.93	27.41	24.95
**31.25**	45.97	42.81	95.24	61.78	40.84	34.92	30.88
**15.6**	57.20	51.56	99.71	79.39	49.60	44.73	36.78
**7.8**	74.19	69.37	100	92.64	70.38	65.46	42.97
**3.9**	88.43	86.02	100	98.25	81.49	79.25	51.36
**2**	95.72	93.88	100	100	92.63	90.48	59.28
**1**	99.34	97.40	100	100	96.75	96.23	72.83
**0**	100	100	100	100	100	100	100

**Table 6:** Cytotoxicity of compounds **15**, **16**, **17**, **18**, **19**, **20** and Doxorubicin against breast carcinoma cell line MCF-7

Concentration µg/mL	Viability (%)/compound						
	**15**	**16**	**17**	**18**	**19**	**20**	Doxorubicin
**500**	8.76	5.28	7.95	6.17	5.21	8.25	1.51
**250**	13.81	13.96	21.34	13.92	11.36	16.37	2.36
**125**	26.92	21.47	30.67	21.40	20.84	28.72	3.21
**62.5**	37.24	32.68	38.72	28.57	27.30	41.89	5.07
**31.25**	48.17	36.43	51.90	36.71	35.26	49.78	6.93
**15.6**	60.95	42.90	64.18	48.86	39.48	80.93	15.46
**7.8**	76.43	51.72	72.37	69.52	46.29	92.34	19.89
**3.9**	89.71	69.37	88.45	81.94	54.68	97.28	24.98
**2**	97.02	83.19	92.32	90.68	69.31	100	31.69
**1**	100	92.48	97.45	96.25	80.47	100	40.17
**0**	100	100	100	100	100	100	100

The resulting data of the 50% inhibition concentration (IC_50_) summarized in Table 7 showed that, the synthesized compounds have different activity against hepatocellular carcinoma cell line HePG2 and breast cancer cell line MCF-7.

**Table 7:** IC_50_ of the tested compounds against hepatocellular carcinoma cell line HePG2 and breast cancer cell line MCF-7

Compound no.	HepG-2 cell line	MCF-7 cell line
**1**	14.7	26.3
**2**	102	115
**3**	4.54	6.58
**4**	15.4	14.9
**5**	3.42	3.98
**6**	229	276
**7**	51.5	61.3
**8**	14.1	19.1
**9**	14.9	25.6
**10**	13.5	18.4
**11a**	243	249
**11b**	32.2	53.3
**12**	10.9	15.4
**13**	12.9	13.6
**14**	2.33	4.53
**15**	17.3	29
**16**	7.19	9.32
**17**	26.6	35.8
**18**	9.94	15.2
**19**	3.78	6.08
**20**	28.5	31.1
Doxorubicin	0.85	0.35

## Conclusion

A novel series of substituted 2-thiohydantoin with incorporated benzoimidazole, pyrazole, triazole and/or benzoxazole moieties has been synthesized. The structures of these compounds were confirmed by IR, ^1^HNMR, ^13^CNMR, MS and elemental analysis. The bioassay results revealed that, Compound **14** has the best activity against HePG2 cell line (IC_50_ = 2.33 μg/mL), while compound **5** has the best activity against MCF-7 cell line (IC_50_ = 3.98 μg/mL). Structure and biological activity relationship showed that, the activity of thiohydantoin diverse with the substituents on it, where introducing active groups such as, CH_3_CO, OH, OCH_3_, OC_2_H_5_, =CH–OC_2_H_5_ and the presence of benzoimidazole, pyrazolone, pyrazole carbonitrile and triazine moieties enhanced the activity of thiohydantoin.

## Experimental section

Melting points were measured using electrothermal digital melting points apparatus and are uncorrected. IR (infrared) spectra were recorded on NICOLET (iS50 FT-IR) spectrometer using KBr pellets. ^1^H and ^13^C NMR (nuclear magnetic resonance) were recorded on a Bruker AS 850 TM spectrometer at 850 MHz and chemical shifts were given with respect to TMS (tetramethylsilane). Mass (MS) spectra were recorded on GC/MS with CI (chemical ionization) and a Hewlett-Packard MS Engine Thermospray and ionization by electron impact to (70 eV). Microanalysis was conducted using elemental analyzer 106.

### Synthesis of (E)-3-[1-(4-bromophenyl)ethylideneamino]-2-thioxoimidazolidin-4-one **1**

A mixture of (*E*)-2-[1-(4-bromophenyl)ethylidene]hydrazinecarbothioamide (0.01 mol) and ethyl chloroacetate (0.01 mol) in ethanol (50 mL) in the presence of fused sodium acetate (0.03 mol) was heated under reflux for 2 h, then cooled and poured into water. The solid formed was filtered off, washed with water, dried and purified from ethanol to give **1**.

### Synthesis of (*E*)-ethyl 2-{3-[1-(4-bromophenyl)ethylideneamino]-4-oxo-2-thioxoimidazolidin-1-yl}acetate **2**

A mixture of **1** (0.01 mol), ethyl chloroacetate (0.01 mol) and anhydrous potassium carbonate (0.015 mol) in 20 mL ethanol was stirred under reflux for 6 h. The reaction mixture was poured into an ice-water mixture. The solid product separated was filtered off, washed with water, dried and crystallized from ethanol to give **2** as pale yellow crystals, in yield 92%, m.p 78–80 °C. ^1^H-NMR (DMSO-*d*_*6*_): *δ* = 7.79 (d, 2H, *J* = 8.5 Hz, 2CH), 7.65 (d, 2H, *J* = 8.5 Hz, 2H, 2CH), 4.52 (s, 2H, CH_2_), 4.16 (q, 2H, *J* = 6.8 Hz, CH_2_), 4.10 (s, 2H, CH_2_), 2.34 (s, 3H, CH_3_) and 1.20 (t, 3H, *J* = 6.8 Hz, CH_3_) ppm. ^13^C-NMR (DMSO-*d*_*6*_): *δ* = 171. 68 (C=S), 166.96, 162.10 (2C=O), 161.40 (C=N), 136.65, 131.46, 128.48, 123.66 (C-aromatic), 61.23 (CH_2_), 58.94 (CH_2_), 32.11 (CH_2_), 14.33 (CH_3_) and 14.24 (CH_3_) ppm. IR (KBr): 1742, 1711 (2C=O), 1611 (C=N) and 1386 (C=S) cm^−1^. MS: m/z (%): 398 (31), 397 (M^+^, ^79^Br, 100) and 399 (M^+^, ^81^Br, 80). Anal. Calcd. C_15_H_16_BrN_3_O_3_S (398.27): C, 45.24; H, 4.05; Br, 20.06; N, 10.55; S, 8.05;. Found. C, 45.30; H, 4.11; Br, 19.97; N, 10.47; S, 8.10.

### Synthesis of (*E*)-2-{3-[1-(4-bromophenyl)ethylideneamino]-4-oxo-2-thioxoimidazolidin-1-yl}acetic acid **3**

Sodium hydroxide (30 mL/4 N) was added to a solution of **2** (0.01 mol) in ethanol (30 mL), then heated under reflux for 2 h. The reaction mixture was cooled and acidified with 2 N hydrochloric acid. The solid formed was filtered off, washed with water, dried and purified from ethanol to give **3** as pale yellow crystals, in yield 52%, m.p 213–215 °C. ^1^H-NMR (DMSO-*d*_*6*_): *δ* = 9.37 (br. s, 1H, OH), 7.70 (d, 2H, *J* = 8.5 Hz, 2CH), 7.57 (d, 2H, *J* = 8.5 Hz, 2CH), 4.53 (s, 2H, CH_2_), 3.83 (s, 2H, CH_2_) and 2.28 (s, 3H, CH_3_) ppm. ^13^C-NMR (DMSO-*d*_*6*_): *δ* = 173.45 (C=S), 168.83, 165.20 (2C=O), 157.19 (C=N), 131.77, 130.78, 128.70, 126.03 (C-aromatic), 75.1 (CH_2_), 29.81 (CH_2_) and 14.03 (CH_3_) ppm. IR (KBr): 3308 (br. OH), 1725, 1675 (2C=O), 1617 (C=N) and 1393 (C=S) cm^−1^. MS: m/z (%): 370 (4), 369 (M^+^, ^79^Br, 14), 371 (M^+^, ^81^Br, 1), 368 (M^+^ − 1, 34) and 57 (100). Anal. Calcd. C_13_H_13_BrN_3_O_3_S (370.22): C, 42.17; H, 3.27; Br, 21.58; N, 11.35; S, 8.66;. Found. C, 42.27; H, 3.29; Br, 21.71; N, 11.48; S, 8.59.

### Synthesis of (*E*)-2-{3-[1-(4-bromophenyl)ethylideneamino]-4-oxo-2-thioxoimidazolidin-1-yl}acetohydrazide **4**

A mixture of **3** (0.01 mol) and hydrazine hydrate (0.03 mol) in ethanol (20 mL), was heated under reflux for 2 h, the reaction mixture was cooled, then poured into ice/water solution and acidified with hydrochloric acid (1 N). The resulting solid was filtered off, washed with water, dried and crystallized from ethanol to give **4** as white crystals, in yield 85%, m.p 196–198 °C. ^1^H-NMR (DMSO-*d*_*6*_): *δ* = 10.25 (s, 1H, NH), 8.30 (s, 2H, NH_2_), 7.90 (d, 2H, *J* = 8.5 Hz, 2CH), 7.78 (d, 2H, *J* = 8.5 Hz, 2CH), 4.43 (s, 2H, CH_2_), 4.07 (s, 2H, CH_2_) and 2.35 (s, 3H, CH_3_) ppm. ^13^C-NMR (DMSO-*d*_*6*_): *δ* = 171.67 (C=S), 166.96, 165.43 (2C=O), 161.36 (C=N), 136.69, 131.45, 128.44, 123.67 (C-aromatic), 61.28, 44.32 (2CH_2_), and 14.38 (CH_3_). IR (KBr): 3407 (NH), 3225, 3190 (NH_2_), 1716, 1669 (2C=O), 1584 (C=N) and 1393 (C=S) cm^−1^. MS: m/z (%): 384 (34), 383 (M^+^, ^79^Br, 5), 385 (M^+^, ^81^Br, 15) and 381 (100). Anal. Calcd. C_13_H_14_BrN_5_O_2_S (384.25): C, 40.63; H, 3.67; Br, 20.79; N, 18.23; S, 8.34;. Found. C, 40.69; H, 3.56; Br, 20.75; N, 18.35; S, 8.27.

#### Typical procedure for syntheses of compounds **5**–**7**

A mixture of compound **4** (0.01 mol) and an equimolar amount of ethyl acetoacetate or acetylacetone or ethyl cyanoacetate (or diethyl malonate) was refluxed in 10 mL of acetic acid for 5 h. The product formed after cooling was filtered off, washed with water, dried and crystallized with acetic acid to give compounds **5**, **6**, and **7**, respectively.

##### (E)-1-(2-{3-[1-(4-bromophenyl)ethylideneamino]-4-oxo-2-thioxoimidazolidin-1-yl}acetyl)-3-methyl-1H-pyrazol-5(4H)-one **5**

Yellow crystals, in yield 74%, m.p 177–179 °C. ^1^H-NMR (DMSO-*d*_*6*_): *δ* = 7.74 (d, 2H, *J* = 8.5 Hz, 2CH), 7.65 (d, 2H, *J* = 8.5 Hz, 2CH), 4.52 (s, 2H, CH_2_), 4.09 (s, 2H, CH_2_), 3.89 (s, 2H, CH_2_), 2.34 (s, 3H, CH_3_) and 1.89 (s, 3H, CH_3_) ppm. ^13^C-NMR (DMSO-*d*_*6*_): *δ* = 171.73 (C=S), 168.02, 167.01, 164.60 (3C=O), 161.47, 161.29 (C=N), 136.68, 131.43, 128.53, 123.71 (C-aromatic), 61.33, 43.80, 32.16 (3CH_2_) and 20.47, 14.62 (2CH_3_). IR (KBr): 1716, 1741 (C=O), 1605, 1589 (2C=N) and 1386 (C=S) cm^−1^. MS: m/z (%): 450 (13), 449 (M^+^, ^79^Br, 23), 451 (M^+^, ^81^Br, 7) and 427 (100). Anal. Calcd. C_17_H_16_BrN_5_O_3_S (450.31): C, 45.34; H, 3.58; Br, 17.74; N, 15.55; S, 7.12;. Found. C, 45.38; H, 3.62; Br, 17.83; N, 15.49; S, 7.15.

##### (E)-3-[1-(4-bromophenyl)ethylideneamino]-1-{2-(3,5-dimethyl-1H-pyrazol-1-yl)-2-oxoethyl}-2-thioxoimidazolidin-4-one **6**

Black crystals, in yield 82%, m.p 83–85 °C. ^1^H-NMR (DMSO-*d*_*6*_): *δ* = 7.89–7.65 (m, 5H, Ar–H), 4.52 (s, 2H, CH_2_), 4.10 (s, 2H, CH_2_), 2.57 (s, 3H, CH_3_), 2.34 (s, 3H, CH_3_) and 1.82 (s, 3H, CH_3_) ppm. ^13^C-NMR (DMSO-*d*_*6*_): *δ* = 170.05 (C=S), 166.18, 164.60 (2C=O), 160.36, 160.19 (2C=N), 138.16, 138.08, 132.57, 132.14, 128.20, 123.43 (C-aromatic) and 58.75, 31.19 (2CH_2_), 19.83, 16.34, 14.52 (3CH_3_) ppm. IR (KBr): 1722 (C=O), 1607, 1585 (2C=N) and 1370 (C=S) cm^−1^. MS: m/z (%): 448 (12), 447 (M^+^, ^79^Br, 5) and 450 (^81^Br, M^+^ + 1, 14), 76 (100). Anal. Calcd. C_18_H_18_BrN_5_O_2_S (448.34): C, 48.22; H, 4.05; Br, 17.82; N, 15.62; S, 7.15. Found. C, 48.31; H, 3.95; Br, 17.95; N, 15.70; S, 7.21.

##### (E)-1-(2-{3-[1-(4-bromophenyl)ethylideneamino]-4-oxo-2-thioxoimidazolidin-1-yl}acetyl)pyrazolidine-3,5-dione **7**

Pale yellow crystals, in yield 69%, m.p 228–230 °C. ^1^H-NMR (DMSO-*d*_*6*_): *δ* = 10.81 (s, 1H, NH), 7.66 (d, 2H, *J* = 8.5 Hz, 2CH), 7.63 (d, 2H, *J* = 8.5 Hz, 2CH), 4.52 (s, 2H, CH_2_), 4.10 (s, 2H, CH_2_), 4.03 (s, 2H, CH_2_) and 2.34 (s, 3H, CH_3_) ppm. ^13^C-NMR (DMSO-*d*_*6*_): *δ* = 174.12 (C=S), 169.25, 166.78, 165.44, 163.99 (4C=O), 159.89 (C=N), 132.41, 131.43, 128.45, 123.98 (C-aromatic), 58.55, 44.32, 31.73 (3CH_2_) and 16.67 (CH_3_) ppm. IR (KBr): 3196 (NH), 1717 (C=O), 1603 (C=N) and 1392 (C=S) cm^−1^. MS: m/z (%): 453 (6), 452 (M^+^, ^79^Br, 11) and 454 (M^+^, ^81^Br, 10) and 101 (100). Anal. Calcd. C_16_H_14_BrN_5_O_4_S (452.28): C, 42.49; H, 3.12; Br, 17.67; N, 15.48; S, 7.09;. Found. C, 42.42; H, 3.15; Br, 17.60; N, 15.40; S, 7.11.

### Synthesis of (*E*)-5-amino-1-(2-{3-[1-(4-bromophenyl)ethylideneamino]-4-oxo-2-thioxoimidazolidin-1-yl}acetyl)-1*H*-pyrazole-4-carbonitrile **8**

To compound **4** (0.01 mol) dissolved in 50 mL absolute ethanol was added slowly with shaking, ethoxymethylenemalononitrile (0.01 mol), after addition of about half of the quantity, the solution was carefully heated to boiling. The remaining ethoxymethylenemalononitrile was added, at such a rate to maintain gentle boiling of the solution, after all the ethoxymethylenemalononitrile had been added, the solution was gently boiled for an additional 30 min and finally was set aside overnight in the refrigerator. The product formed was filtered off, dried and crystallized from ethanol to give **8** as yellow crystals, in yield 84%, m.p 198–200 °C. ^1^H-NMR (DMSO-*d*_*6*_): *δ* = 7.79–7.62 (m, 5H, Ar–H, N = CH), 4.94 (s, 2H, NH_2_), 4.68 (s, 2H, CH_2_), 4.55 (s, 2H, CH_2_) and 2.35 (s, 3H, CH_3_) ppm. ^13^C-NMR (DMSO-*d*_*6*_): *δ* = 172.00 (C=S), 165.21, 162.77 (2C=O), 161.06, 160.87 (2C=N), 119.51 (CN), 136.79, 131.46, 131.39, 128.45, 128.26 (C-aromatic), 43.87, 32.30 (2CH_2_) and 14.49 (CH_3_) ppm. IR (KBr): 3289, 3194 (NH_2_), 2203 (CN), 1726, 1660 (C=O), 1606 (C=N) and 1388 (C=S) cm^−1^. MS: m/z (%): 460 (5), 459 (M^+^, ^79^Br, 11), 461 (M^+^, ^81^Br, 10) and 383 (100). Anal. Cald. C_17_H_14_BrN_7_O_2_S (460.31): C, 44.36; H, 3.07; Br, 17.36; N, 21.30; S, 6.97. Found. C, 44.52; H, 3.13; Br, 17.48; N, 21.27; S, 6.83.

### Synthesis of (*E*)-2-(2-{3-[1-(4-bromophenyl)ethylideneamino]-4-oxo-2-thioxoimidazolidin-1-yl}acetyl)-*N*-phenylhydrazinecarbothioamide **9**

A mixture of **4** (0.01 mol) and phenyl isothiocyanate (0.01 mol) in dimethylformamide (25 mL) was stirred under reflux for 5 h. The reaction mixture then cooled to room temperature, poured into ice water, then acidified with dilute hydrochloric acid. The resulting solid was filtered off, washed with water, dried and purified by crystallization from ethanol to give **9** as orange crystals, in yield 89%, m.p 68–70 °C. ^1^H-NMR (DMSO-*d*_*6*_): *δ* = 11.06 (s, 1H, NH), 8.54 (s, 1H, NH), 9.86 (s, 1H, NH), 7.71–7.24 (m, 9H, Ar–H), 4.52 (s, 2H, CH_2_), 4.16 (s, 2H, CH_2_) and 2.33 (s, 3H, CH_3_) ppm. IR (KBr): 3189 (NH), 1653, 1723 (C=O), 1607 (C=N) and 1385 (C=S) cm^−1^. MS: m/z (%): 519 (3), 518 (M^+^, ^79^Br, 6), 520 (M^+^, ^81^Br, 5) and 438 (100). Anal. Calcd. C_20_H_19_BrN_6_O_2_S_2_ (519.44): C, 46.24; H, 3.69; Br, 15.38; N, 16.18; S, 12.35. Found. C, 46.19; H, 3.59; Br, 15.52; N, 16.25; S, 12.39.

### Synthesis of (*E*)-3-[1-(4-bromophenyl)ethylideneamino]-1-{(4-phenyl-5-thioxo-4,5-dihydro-1*H*-1,2,4-triazol-3-yl)methyl}-2-thioxoimidazolidin-4-one **10**

A solution of **9** (0.01 mol) in ethanol (30 mL) was added with sodium hydroxide (30 mL/1 N), then heated under reflux for 4 h. The reaction mixture was cooled and acidified with diluted hydrochloric acid. The solid formed was filtered off, washed with water, dried and purified from ethanol to give **10** as white crystals, in yield 66%, m.p 48–50 °C. ^1^H-NMR (DMSO-*d*_*6*_): *δ* = 11.05 (s, 1H, NH), 7.79–7.35 (m, 9H, Ar–H), 4.53 (s, 2H, CH_2_), 4.04 (s, 2H, CH_2_) and 2.26 (s, 3H, CH_3_) ppm. ^13^C-NMR (DMSO-*d*_*6*_): *δ* = 177.90, 172.27 (2C=S), 166.25 (C=O), 159.16, 159.02 (2C=N), 138.66, 137.83, 128.92, 128.51, 125.16, 124.70, 123.15, 121.76 (C-aromatic), 65.27, 30.77 (2CH_2_) and 14.03 (CH_3_) ppm. IR (KBr): 3408 (NH), 1715 (C=O), 1604 (C=N) and 1385 (C=S) cm^−1^. MS: m/z (%): 501 (6), 500 (M^+^, ^79^Br, 7), 502 (M^+^, ^81^Br, 3). Anal. Calcd. C_20_H_17_BrN_6_OS_2_ (501.42): C, 47.91; H, 3.42; Br, 15.94; N, 16.76; S, 12.79. Found. C, 47.83; H, 3.30, Br, 15.90; N, 16.81; S, 12.83.

#### Syntheses of compounds **11a** and **11b**

A mixture of **4** (0.01 mol), aromatic aldehydes such as, (isonicotinaldehyde and anisaldehyde) (0.01 mol) and piperidine (1 mL) was fused on a hot plate at 100–110 °C for half an hour, then ethanol (25 mL) was added and refluxed for 2 h. The reaction mixture then cooled and acidified with diluted hydrochloric acid. The resulting solid was filtered off, washed with water, dried and purified by crystallization from proper solvent to give **11a**, **b**.

##### (E)-2-{3-[1-(4-Bromophenyl)ethylideneamino]-4-oxo-2-thioxoimidazolidin-1-yl}-*N*′-(pyridin-4-ylmethylene)acetohydrazide **11a**

Pale yellow crystals, in yield 84%, m.p 270–272 °C (benzene). ^1^H-NMR (DMSO-*d*_*6*_): *δ* = 8.76–7.64 (m, 10H, Ar–H, pyridine, =CH, NH), 4.51 (s, 2H, CH_2_), 4.11 (s, 2H, CH_2_) and 2.32 (s, 3H, CH_3_) ppm. ^13^C-NMR (DMSO-*d*_*6*_): *δ* = 172.12 (C=S), 167.32, 163.44 (2C=O), 162.72, 142.32 (2C=N), 140.43, 136.71, 136.26, 131.58, 128.83, 124.15, 123.59 (C-aromatic), 43.71, 32.12 (CH_2_) and 14.38 (CH_3_) ppm. IR (KBr): 3190 (NH), 1678, 1724 (C=O), 1607 (C=N) and 1396 (C=S) cm^−1^. MS: m/z (%): 473 (28), 472 (M^+^, ^79^Br, 74) and 474 (M^+^, ^81^Br, 100). Anal. Calcd. C_19_H_17_BrN_6_O_2_S (473.35): C, 48.21; H, 3.62; Br, 16.88; N, 17.75; S, 6.77. Found. C, 48.29; H, 3.64; Br, 16. 89; N, 17.69; S, 6.70.

##### (E)-2-{3-[1-(4-Bromophenyl)ethylideneamino]-4-oxo-2-thioxoimidazolidin-1-yl}-*N*′-(4-methoxybenzylidene)acetohydrazide **11b**

Pale yellow crystals, in yield 79%, m.p 229–231 °C (EtOH). ^1^H-NMR (DMSO-*d*_*6*_): *δ *= 11.63 (s, 1H, NH), 7.98–7.00 (m, 9H, Ar–H, =CH), 4.46 (s, 2H, CH_2_), 4.08 (s, 2H, CH_2_), 3.87 (s, 3H, OCH_3_) and 2.33 (s, 3H, CH_3_) ppm. ^13^C-NMR (DMSO-*d*_*6*_): *δ* = 172.73 (C=S), 167.10, 162.54 (2C=O), 161.02, 141.99 (2C=N), 141.03, 136.26, 128.45, 128.45, 126.47, 123.46, 121.06, 120.98 (C-aromatic), 56.77 (OCH_3_), 44.49, 32.23 (2CH_2_) and 14.44 (CH_3_) ppm. IR (KBr): 3189 (NH), 1675, 1723 (C=O), 1608 (C=N) and 1395 (C=S) cm^−1^. MS: m/z (%): 502 (38), 501 (M^+^, ^79^Br, 98) and 503 (M^+^, ^81^Br, 100). Anal. Calcd. C_21_H_20_BrN_5_O_3_S (502.38): C, 50.21; H, 4.01; Br, 15.90; N, 13.94; S, 6.38,. Found. C, 50.10; H, 3.87: Br, 16.03; N, 14.02; S, 6.30.

#### Syntheses of compounds **12** and **13**

A mixture of **1** (0.01 mol) and triethyl orthoformate and/or diethyl oxalate (0.01 mol) in xylene (25 mL) in the presence of sodium metal (0.50 g), was heated under reflux for 4 h, then filtered upon hot and the filtrate then concentrated, cooled and the solid formed was filtered off, dried and purified by crystallization from ethanol to give **12** and **13**, respectively.

##### (E)-3-[1-(4-Bromophenyl)ethylideneamino]-5-(ethoxymethylene)-2-thioxoimidazolidin-4-one **12**

Brown crystals, in yield 73%, m.p 181–183 °C. ^1^H-NMR (DMSO-*d*_*6*_): *δ* = 11.42 (s, 1H, NH), 7.74 (d, 2H, *J* = 8.5 Hz, 2CH), 7.66 (d, 2H, *J* = 8.5 Hz, 2CH), 7.08 (s, 1H, =CHO), 4.15 (q, 2H, *J* = 8.6 Hz, CH_2_), 2.29 (s, 3H, CH_3_) and 1.05 (t, 3H, *J* = 6.8 Hz, CH_3_) ppm. ^13^C-NMR (DMSO-*d*_*6*_): *δ* = 172.09 (C=S), 164.58 (C=O), 159.32 (C=N), 135.92 (CH), 133.99, 131.70, 128.24, 123.33 (C-aromatic), 115.45 (HNC=), 65.57 (CH_2_) and 18.28, 14.32 (2CH_3_) ppm. IR (KBr): 3320 (NH), 1751 (C=O), 1621 (C=N) and 1396 (C=S) cm^−1^. MS: m/z (%): 368 (5), 367 (M^+^, ^79^Br, 4), 369 (M^+^, ^81^Br, 3) and 57 (100). Anal. Calcd. C_14_H_14_BrN_3_O_2_S (368.25): C, 45.66; H, 3.83; Br, 21.70; N, 11.41; S, 8.71. Found. C, 45.57; H, 3.72; Br, 21.68; N, 11.48; S, 8.58.

##### (E)-Ethyl 2-{1-[1-(4-bromophenyl)ethylideneamino]-5-oxo-2-thioxoimidazolidin-4-yl}-2-oxoacetate **13**

Yellow crystals, in yield 75%, m.p 161–163 °C. ^1^H-NMR (DMSO-*d*_*6*_): *δ* = 12.00 (s, 1H, NH), 7.74 (d, 2H, *J* = 8.5 Hz, 2CH), 7.66 (d, 2H, *J* = 8.5 Hz, 2CH), 4.43 (s, 1H, CH), 4.20 (q, 2H, *J* = 4.2 Hz, CH_2_), 2.26 (s, 3H, CH_3_) and 1.09 (t, 3H, *J* = 4.2 Hz, CH_3_) ppm. ^13^C-NMR (DMSO-*d*_*6*_): *δ* = 176.02 (C=S), 166.82, 163.64, 163.11 (C=O), 160.13 (C=N), 133.89, 131.40, 128.70, 123.38 (C-aromatic), 72.31 (CH), 56.03 (CH_2_) and 14.44, 13.79 (2CH_3_) ppm. IR (KBr): 3411 (NH), 1716 (C=O), 1762 (C=O ester), 1606 (C=N) and 1389 (C=S) cm^−1^. MS: m/z (%): 412 (6), 411 (M^+^, ^79^Br, 8), 413 (M^+^, ^81^Br, 13) and 75 (100). Anal. Calcd. C_15_H_14_BrN_3_O_4_S (412.26): C, 43.70; H, 3.42; Br, 19.38; N, 10.19; S, 7.78. Found. C, 43.58; H, 3.47; Br, 19.51; N, 10.05; S, 7.65.

#### Syntheses of compounds **14** and **15**

A mixture of **13** (0.01 mol) and *o*-phenylenediamine or 2-aminophenol (0.01 mol) in acetic acid (25 mL) was fused under reflux for 2–3 h, then cooled. The solid formed was filtered off, washed with ethanol, dried and purified by crystallization from ethanol to give **14** and **15**.

##### (E)-5-(1H-benzo[d]imidazole-2-carbonyl)-3-[1-(4-bromophenyl)ethylideneamino]-2-thioxoimidazolidin-4-one **14**

Brown crystals, in yield 63%, m.p 238–240 °C. ^1^H-NMR (DMSO-*d*_*6*_): *δ* = 11.99 (s, 1H, NH), 10.26 (s, 1H, NH), 7.91–7.55 (m, 8H, Ar–H), 4.30 (s, 1H, CH) and 2.28 (s, 3H, CH_3_) ppm. ^13^C-NMR (DMSO-*d*_*6*_): *δ* = 178.97 (C=S), 169.08, 167.10 (2C=O), 160.27, 146.63 (2C=N), 136.89, 135.80, 131.56, 130.22, 128.41, 127.30, 123.38, 122.90 (C-aromatic), 73.3 (CH) and 14.47 (CH_3_) ppm. IR (KBr): 3385, 3305 (2NH), 1704, 1730 (2C=O), 1615 (C=N) and 1393 (C=S) cm^−1^. MS: m/z (%): 456 (4), 455 (M^+^, ^79^Br, 6), 457 (M^+^, ^81^Br, 3) and 339 (100). Anal. Calcd. C_19_H_14_BrN_5_O_2_S (456.32): C, 50.01; H, 3.09; Br, 17.51; N, 15.35; S, 7.03. Found C, 49.91; H, 2.93; Br, 17.49; N, 15.42; S, 7.11.

##### (E)-5-(Benzo[d]oxazole-2-carbonyl)-3-[1-(4-bromophenyl)ethylideneamino]-2-thioxoimidazolidin-4-one **15**

Pale orange crystals, in yield 65%, m.p 103–105 °C. ^1^H-NMR (DMSO-*d*_*6*_): *δ* = 9.87 (s, 1H, NH), 7.89–7.20 (m, 8H, Ar–H), 4.30 (s, 1H, CH) and 2.31 (s, 3H, CH_3_) ppm. IR (KBr): 3198 (NH), 1661, 1705 (2C=O), 1616 (C=N) and 1394 (C=S) cm^−1^. MS: m/z (%): 457 (48), 456 (M^+^, ^79^Br, 39), 458 (M^+^, ^81^Br, 75) and 443 (100). Anal. Calcd. C_19_H_13_BrN_4_O_3_S (457.30): C, 49.90; H, 2.87; Br, 17.47; N, 12.25; S, 7.01. Found. C, 49.81; H, 2.75; Br, 17.62; N, 12.32; S, 6.91.

### Synthesis of (*E*)-1-acetyl-3-[1-(4-bromophenyl)ethylideneamino]-2-thioxoimidazolidin-4-one **16**

A solution of **1** (0.01 mol) in acetic anhydride (25 mL) was heated under reflux for 2 h, then cooled and the resulting solid was collected by filtration, dried and purified by crystallization from benzene to give compound **16**, as white crystals, in yield 88%, m.p 164–166 °C. ^1^H-NMR (DMSO-*d*_*6*_): *δ* = 7.59 (d, 2H, *J* = 8.5 Hz, 2CH), 7.43 (d, 2H, *J* = 8.5 Hz, 2CH), 4.34 (s, 2H, CH_2_), 2.23 (s, 3H, CH_3_) and 2.07 (s, 3H, CH_3_) ppm. ^13^C-NMR (DMSO-*d*_*6*_): *δ* = 172.13 (C=S), 164.75, 163.69 (2C=O), 159.83 (C=N), 137.28, 131.36, 128.42, 122.26 (C-aromatic), 82.16 (CH_2_), 22.16 (CH_3_) and 19.47 (CH_3_) ppm. IR (KBr): 1647, 1716 cm^−1^ (2C=O), 1600 (C=N) and 1395 (C=S) cm^−1^. MS: m/z (%): 354 (22), 353 (M^+^, ^79^Br, 91) and 355 (M^+^, ^81^Br, 100). Anal. Calcd. C_13_H_12_BrN_3_O_2_S (354.22): C, 44.08; H, 3.41; Br, 22.56; N, 11.86; S, 9.05;. Found. C, 43.92; H, 3.45; Br, 22.64; N, 11.93; S, 9.13.

### Synthesis of (*E*)-1,5-diacetyl-3-[1-(4-bromophenyl)ethylideneamino]-2-thioxoimidazolidin-4-one **17**

A mixture of **1** (0.01 mol) and fused sodium acetate (0.02 mol) in acetic anhydride (25 mL) was heated under reflux for 3 h, then cooled and poured into ice-water. The resulting solid was filtered off, washed with water, dried and purified by crystallization from benzene to give **17** as pale yellow crystals, in yield 62%, m.p 183–185 °C. ^1^H-NMR (DMSO-*d*_*6*_): *δ* = 7.54 (d, 2H, *J* = 8.5 Hz, 2CH), 7.43 (d, 2H, *J* = 8.5 Hz, 2CH), 5.08 (s, 1H, CH), 2.25 (s, 3H, CH_3_), 2.23 (s, 3H, CH_3_), 2.18 (s, 3H, CH_3_) ppm. ^13^C-NMR (DMSO-*d*_*6*_): *δ* = 169.56 (C=S), 167.85, 164.89, 163.14 (3C=O), 159.85 (C=N), 137.56, 131.42, 128, 28, 122.36 (C-aromatic), 78.00 (CH), 22.48, 22.20 and 18.58 (3CH_3_). IR (KBr): 1646, 1723 and 1752 (3C=O), 1601 (C=N) and 1397 (C=S) cm^−1^. MS: m/z (%): 396 (4), 395 (M^+^, ^79^Br, 18), 397 (M^+^, ^81^Br, 16) and 313 (100). Anal. Calcd. C_15_H_14_BrN_3_O_3_S (396.26): C, 45.47; H, 3.56; Br, 20.16; N, 10.60; S, 8.09. Found. C, 45.38; H, 3.59; Br, 20.12; N, 10.70; S, 7.98.

### Synthesis of (*Z*)-1-acetyl-3-[(*E*)-1-(4-bromophenyl)ethylideneamino]-5-(4-hydroxy-3-methoxybenzylidene)-2-thioxoimidazolidin-4-one **18**

A mixture of **16** (0.01 mol), vanillin (0.01 mol) and piperidine (1 mL) was fused on a hot plate at 100–110 °C for half an hour, then ethanol (25 mL) was added and refluxed for 2 h. The reaction mixture then cooled and acidified with diluted hydrochloric acid. The resulting solid was filtered off, washed with water, dried and purified by crystallization from EtOH to give **18** as yellow crystals, in yield 73%, m.p 78–80 °C. ^1^H-NMR (DMSO-*d*_*6*_): *δ* = 10.26 (br.s, 1H, OH), 7.61–6.95 (m, 8H, Ar–H, CH olefinic), 3.82 (s, 3H, OCH_3_), 2.32 (s, 3H, CH_3_) and 2.03 (s, 3H, CH_3_) ppm. IR (KBr): 3395–3340 (OH), 1751, 1706 (2C=O) groups, 1616 (C=N) and 1396 (C=S) cm^−1^. MS: m/z (%): 488 (11), 487 (M^+^, ^79^Br, 41), 489 (M^+^, ^81^Br, 39) and 430 (100). Anal. Calcd. C_21_H_18_BrN_3_O_4_S (488.35): C, 51.65, H, 3.72; Br, 16.36; N, 8.60; S, 6.57. Found. C, 51.73; H, 3.73; Br, 16.28; N, 8.55; S, 6.50.

### Synthesis of (*E*)-3-[1-(4-bromophenyl)ethylideneamino]-1-[(diethylamino)methyl]-2-thioxoimidazolidin-4-one **19**

To a solution of **1** (0.01 mol) soluble in 50 mL ethanol was added, a mixture of secondary amines (0.01 mol) (diethylamine) and aqueous formaldehyde 37% (1.25 mL) dissolved in 10 mL ethanol, drop wise throw 30 min, then stirred at room temperature for 3 h. Finally refrigerated for 48 h to form crystals. The solid formed was filtered off and crystallized from ethanol to give compound **19** as pale yellow crystals, in yield 70%, m.p 122–124 °C. ^1^H-NMR (DMSO-*d*_*6*_): *δ* = 7.75 (d, 2H, *J* = 8.5 Hz, 2CH), 7.71 (d, 2H, *J* = 8.5 Hz, 2CH), 4.55 (s, 2H, CH_2_), 3.89 (s, 2H, CH_2_), 2.80 (q, 4H, *J* = 6.8 Hz, 2CH_2_), 2.29 (s, 3H, CH_3_) and 1.08 (t, 6H, *J* = 6.8 Hz, 2CH_3_) ppm. IR (KBr): 2820, 2854 (C-aliphatic), 2970 (C-aromatic), 1720 (C=S), 1612 (C=N) and 1384 (C=S) cm^−1^. MS: m/z (%): 397 (10), 396 (M^+^, ^79^Br, 15), 398 (M^+^, ^81^Br, 7) and 298 (100). Anal. Calcd. C_16_H_21_BrN_4_OS (397.33): C, 48.37; H, 5.33; Br, 20.11; N, 14.10; S, 8.07. Found. C, 48.30; H, 5.38; Br, 20.23; N, 13.98; S, 8.14.

### Synthesis of (1*E*,2*E*)-1,2-bis[1-(4-bromophenyl)ethylidene]hydrazine **20**

A mixture of **1** (0.01 mol) and hydrazine hydrate (0.03 mol) was fused on a hot plate at 100–120 °C for half an hour, then adding ethanol (25 mL). The reaction mixture was heated under reflux for 2 h, then poured into ice water and acidified with hydrochloric acid (1 N). The crude product obtained was filtered off, washed with water, dried and purified by crystallization from ethanol to give **20** as white crystals, in yield 78%, m.p 84–86 °C. ^1^H-NMR (DMSO-*d*_*6*_): *δ* = 7.55 (d, 4H, *J* = 8.5 Hz, 4CH), 7.47 (d, 4H, *J* = 8.5 Hz, 4CH) and 2.00 (s, 6H, CH_3_) ppm. ^13^C-NMR (DMSO-*d*_*6*_): *δ* = 140.82 (2C=N), 139.04, 131.00, 126.77, 120.01 (C-aromatic) and 11.19 (2CH_3_) ppm. MS: m/z (%): 394 (33), 393 (M^+^, ^79^Br, 9), 395 (M^+^, ^81^Br, 8) and 55 (100). Anal. Calcd. C_16_H_14_ Br_2_N_2_ (394.10): C, 48.76; H, 3.58; Br, 40.55; N, 7.11. Found. C, 48.82; H, 3.62; Br, 40.39; N, 7.05.

#### Cytotoxicity Assay

In 96-well plate, the cells were seeded at a cell concentration of 1 × 10^4^ cells per well in 100 µL of growth medium. Different concentrations from the tested sample in fresh medium were added after 24 h of seeding. The tested compounds underwent serial two-fold dilutions, then added to confluent cell monolayers dispensed into 96-well, flat-bottomed microtiter plates (Falcon, NJ, USA) using a multichannel pipette. For a period of 48 h, the microtiter plates were incubated in a humidified incubator with 5% CO_2_ at 37 °C. For each concentration of the samples, three wells were used. Control cells were incubated without test sample and with or without DMSO. After incubation of the cells at 37 °C, various concentrations of the sample were added, and the incubation was continued for 24 h and viable cells yield was determined by a colorimetric method.

In brief, media were aspirated and the crystal violet solution (1%) was added to each well for at least 30 min after the end of the incubation period. All excess stain is removed, where the plates were rinsed using tap water. To all wells, glacial acetic acid (30%) was then added and mixed thoroughly, and then the absorbance of the plates was measured after gently shaken on the Microplate reader (TECAN, Inc.), using a test wavelength of 490 nm. All results were corrected for background absorbance detected in wells without added stain. Treated samples were compared with the cell control in the absence of the tested compounds. All experiments were carried out in triplicate. The cell cytotoxic effect of each tested compound was calculated. The optical density was measured with the microplate reader (Sunrise, TECAN, Inc, USA) to determine the number of viable cells and the percentage of viability was calculated as [1 − (ODt/ODc)] × 100% where ODt is the mean optical density of wells treated with the tested sample and ODc is the mean optical density of untreated cells. The relation between surviving cells and drug concentration is plotted to get the survival curve of each tumor cell line after treatment with the specified compound. The 50% inhibitory concentration (IC_50_), the concentration required to cause toxic effects in 50% of intact cells, was estimated from graphic plots of the dose–response curve for each conc. using Graphpad Prism software (San Diego, CA. USA).

## Additional file

**Additional file 1.** Supplimentary materials (spectroscopic data).
