# Supplementary material for: Design, synthesis and evaluation of anticancer activity of novel 2-thioxoimidazolidin-4-one derivatives bearing pyrazole, triazole and benzoxazole moieties
Source: Chem Cent J. 2018 May 9;12:51. doi: 10.1186/s13065-018-0418-1 (PMC5940965; doi:10.1186/s13065-018-0418-1)
Supplement: Supplementary file 1 — Additional file 1. Supplimentary materials (spectroscopic data). [file 13065_2018_418_MOESM1_ESM.docx]

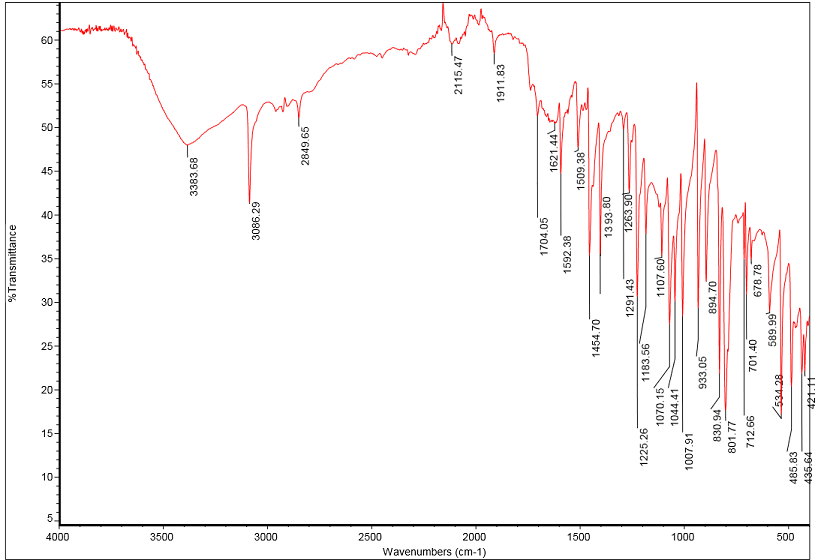


Figure S**1**: IR of compound **1**.


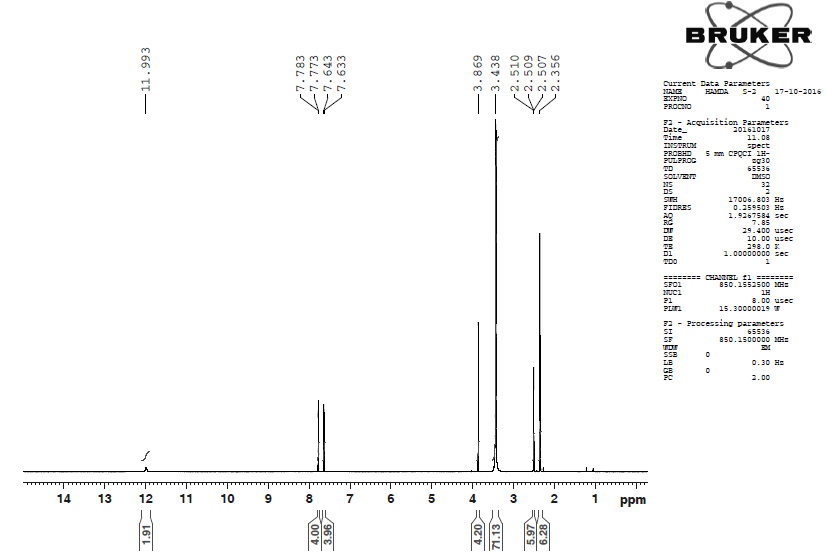


Figure S**2**: ^1^HNMR spectrum of compound **1**.


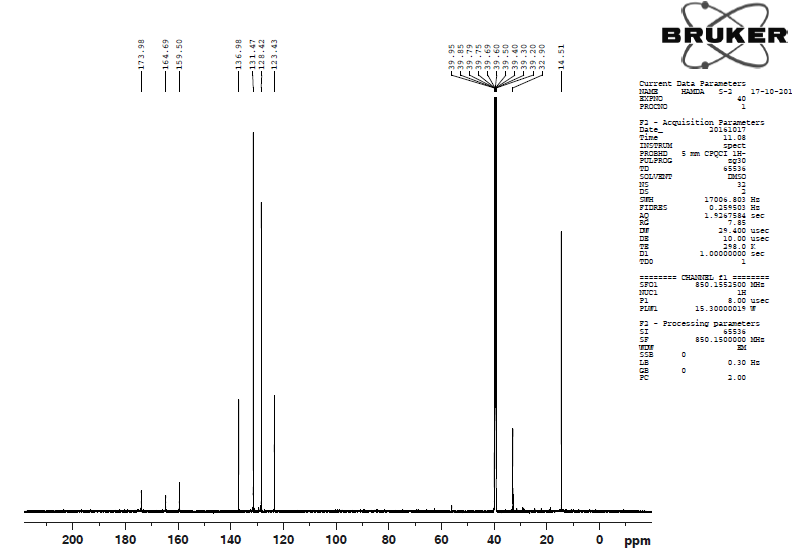


Figure S**3**: ^13^CNMR spectrum of compound **1**


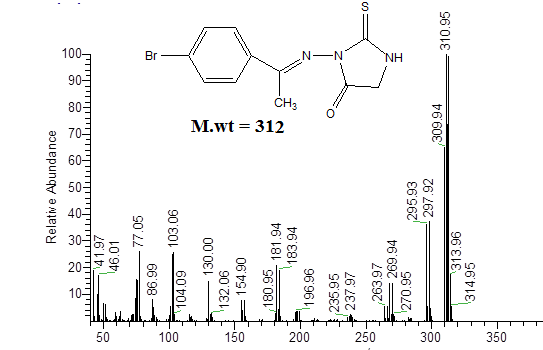


Figure S**4**: Mass spectrum (70 ev) of compound **1**


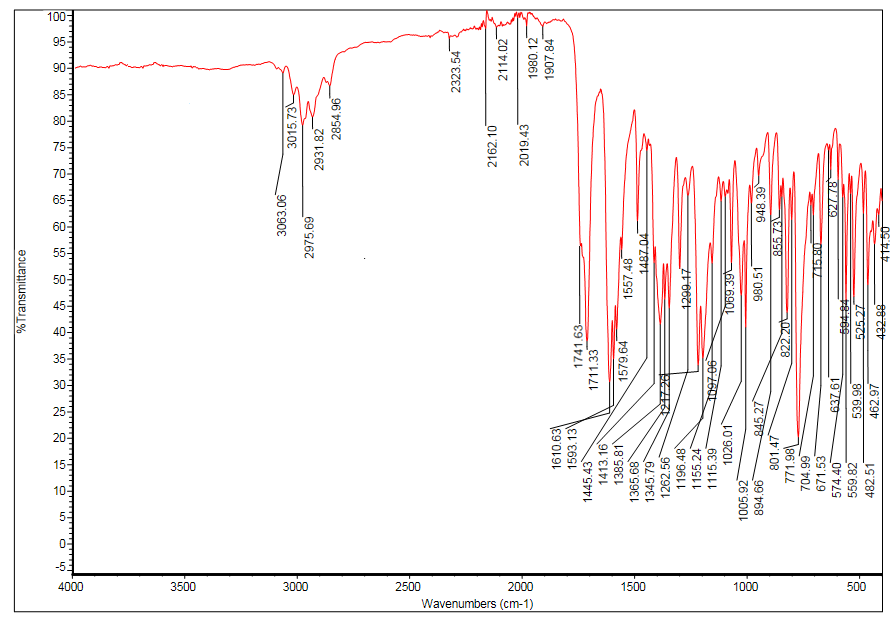


Figure S**5**: IR of compound **2**


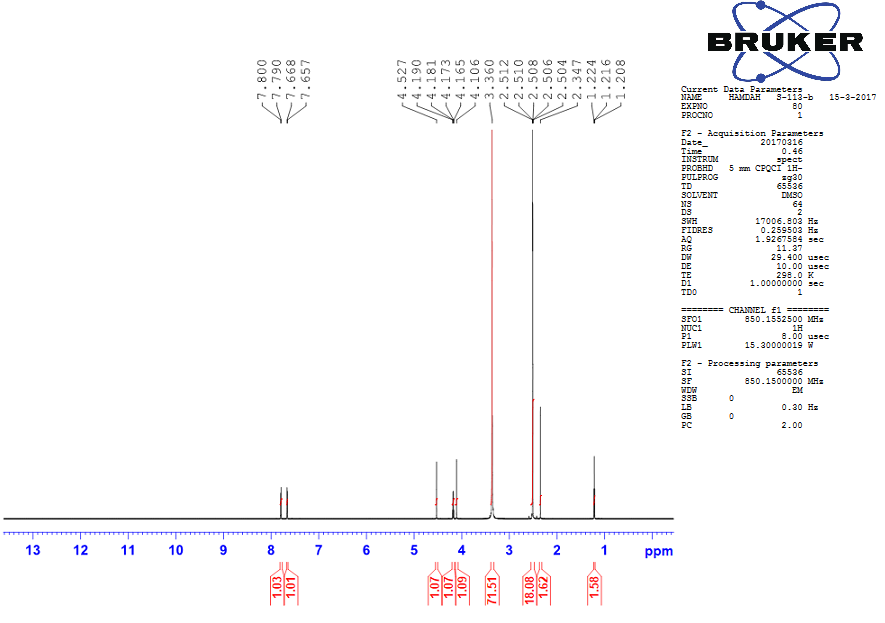


Figure S**6**: ^1^HNMR spectrum of compound **2**

**
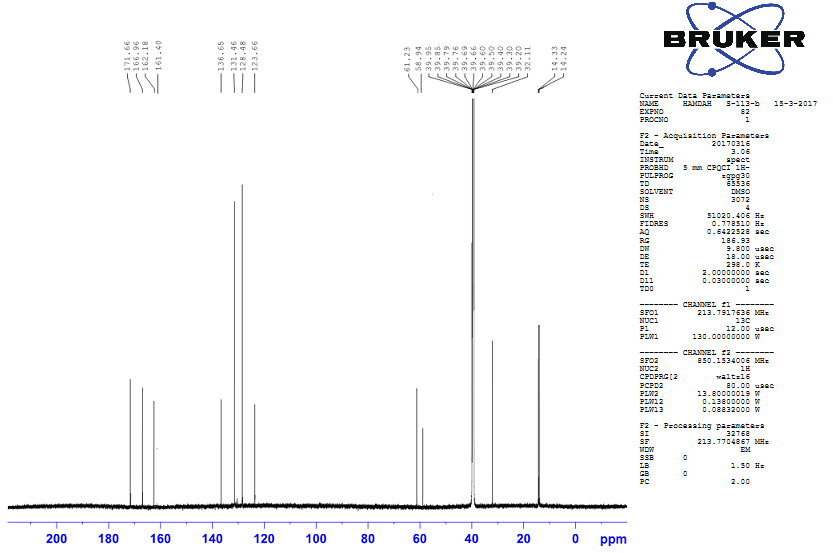
**

Figure S**7**: ^13^CNMR spectrum of compound **2**


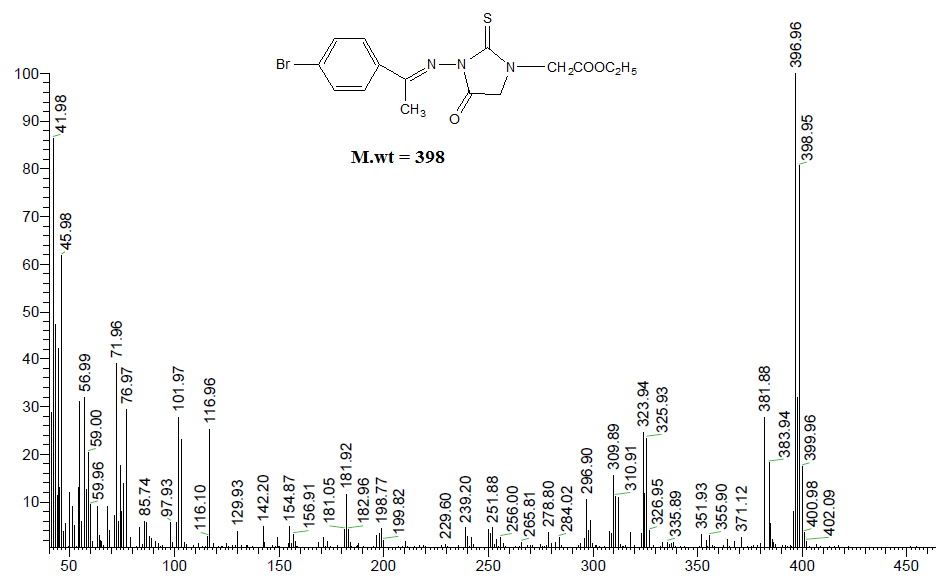


Figure S**8**: Mass spectrum (70 ev) of compound **2**


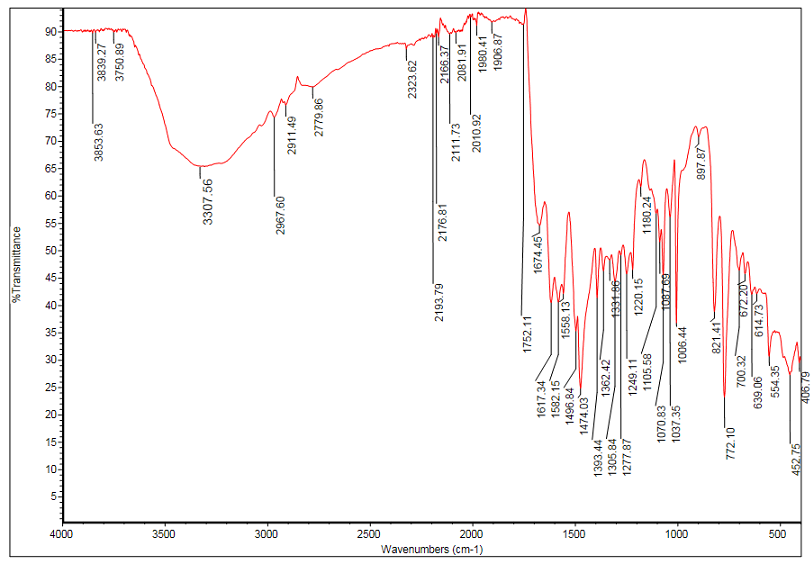


Figure S**9**: IR of compound **3**


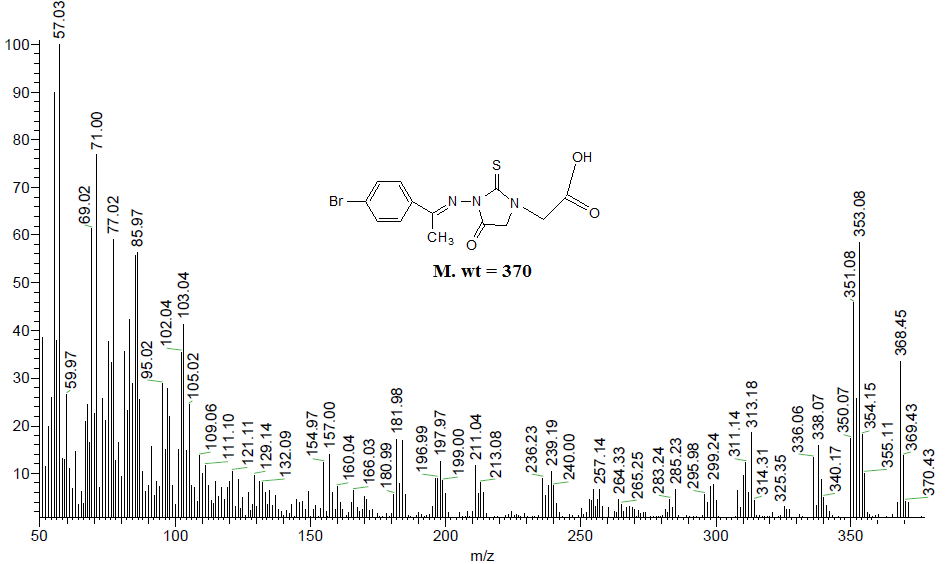


Figure S**10**: Mass spectrum (70 ev) of compound **3**


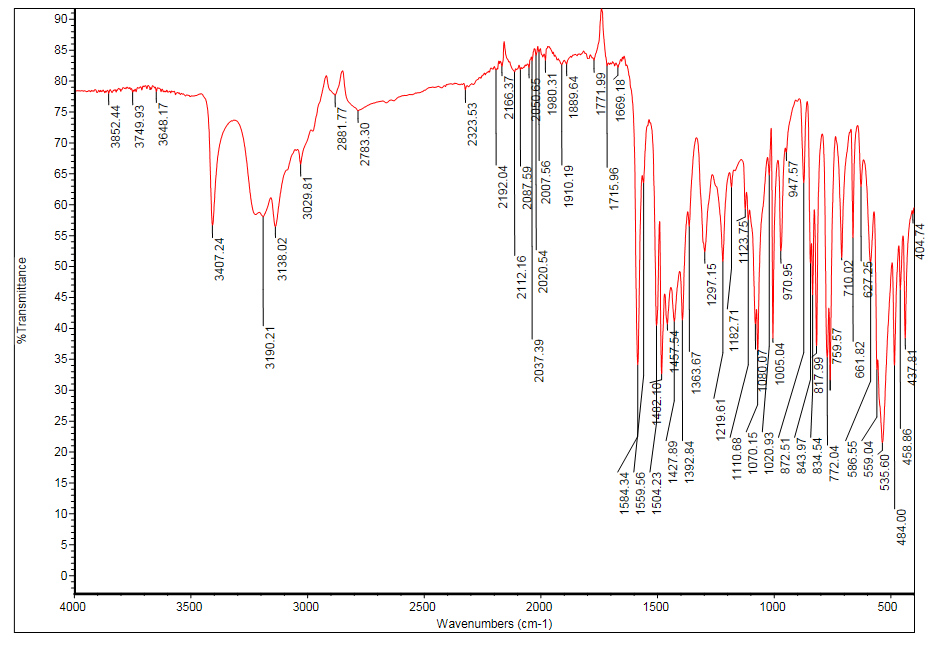
Figure S**11**: IR of compound **4**


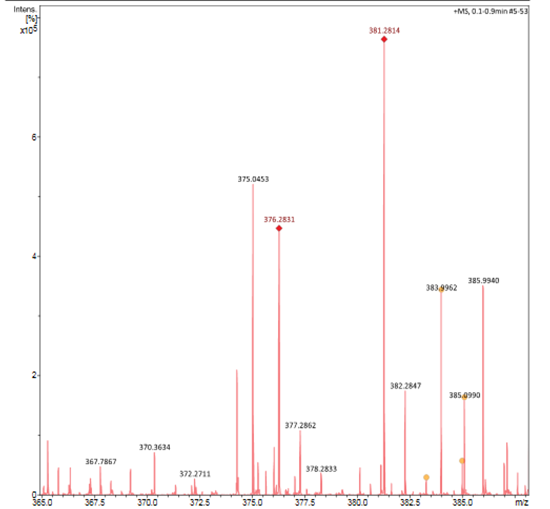


Figure S**12**: Mass spectrum of compound **4**


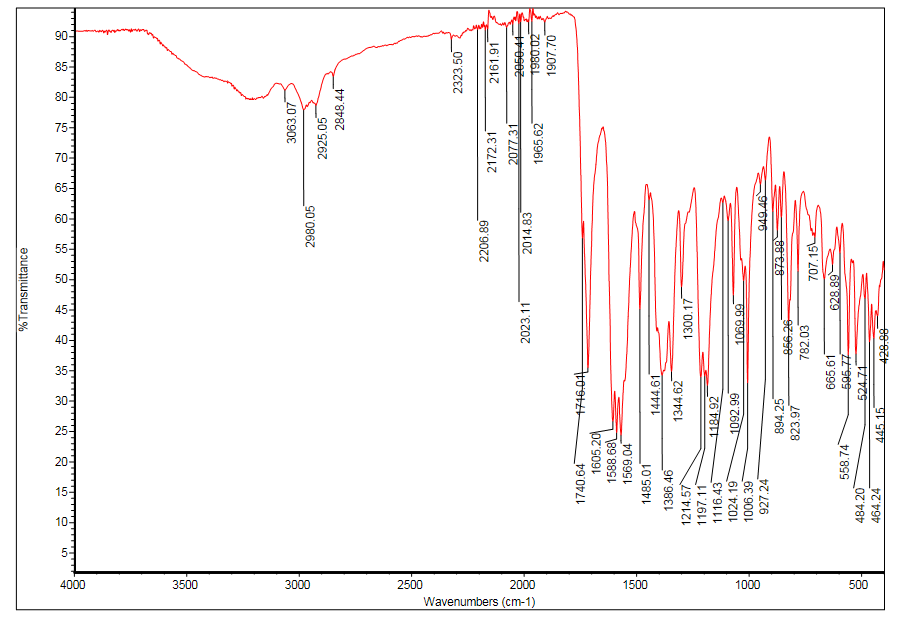


Figure S**13**: IR of compound **5**.


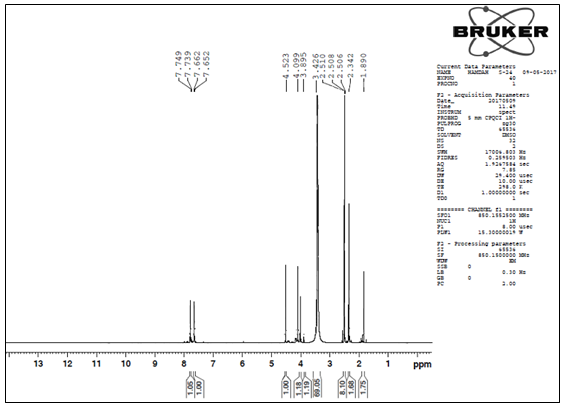


Figure S**14**: ^1^HNMR spectrum of compound **5**.


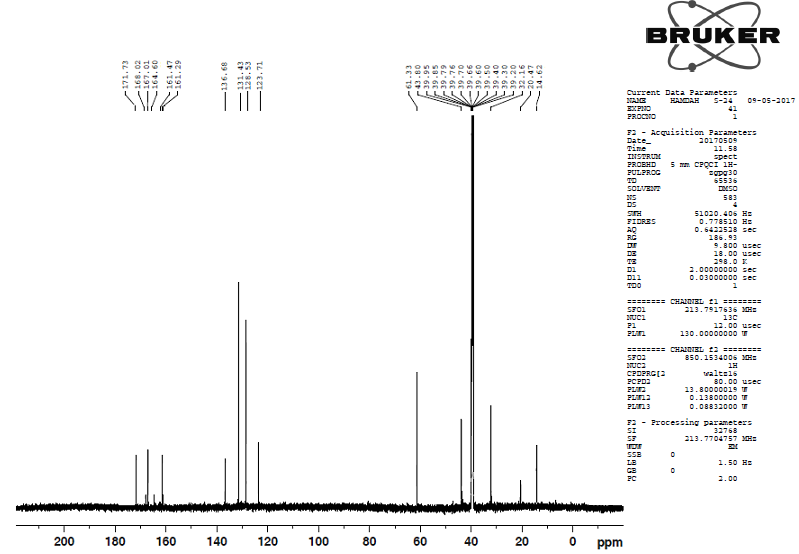


Figure S**15**: ^13^CNMR spectrum of compound **5**


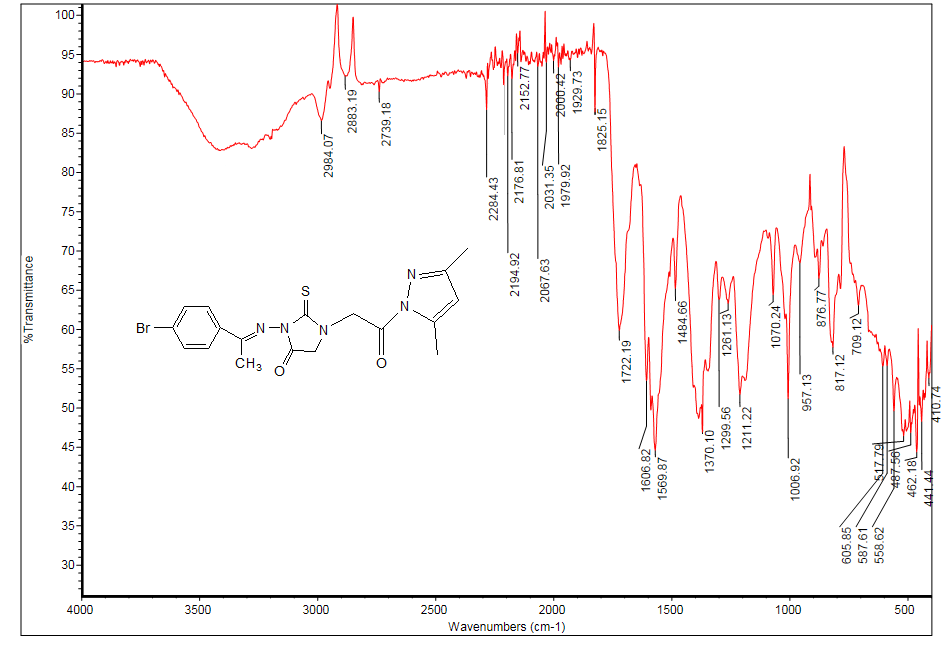
Figure S**16**: IR of compound **6**.


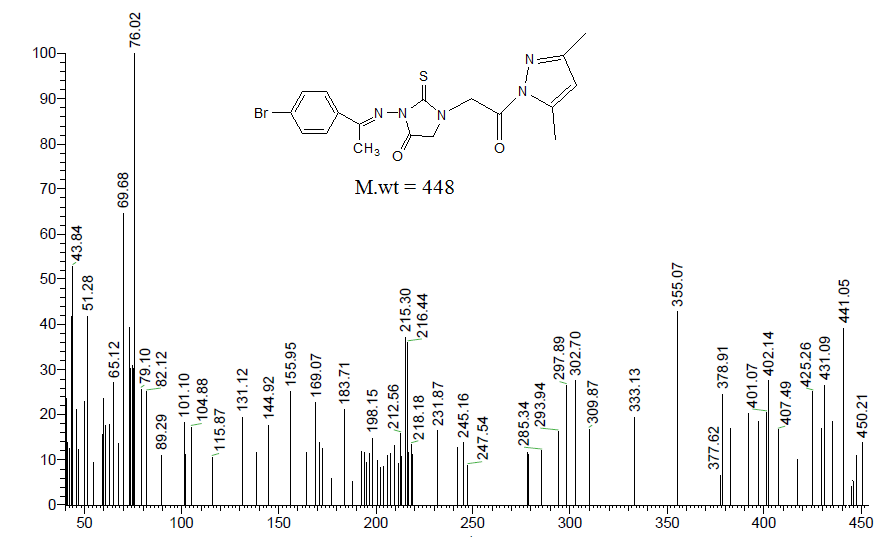


Figure S**17**: Mass spectrum (70 ev) of compound **6**.


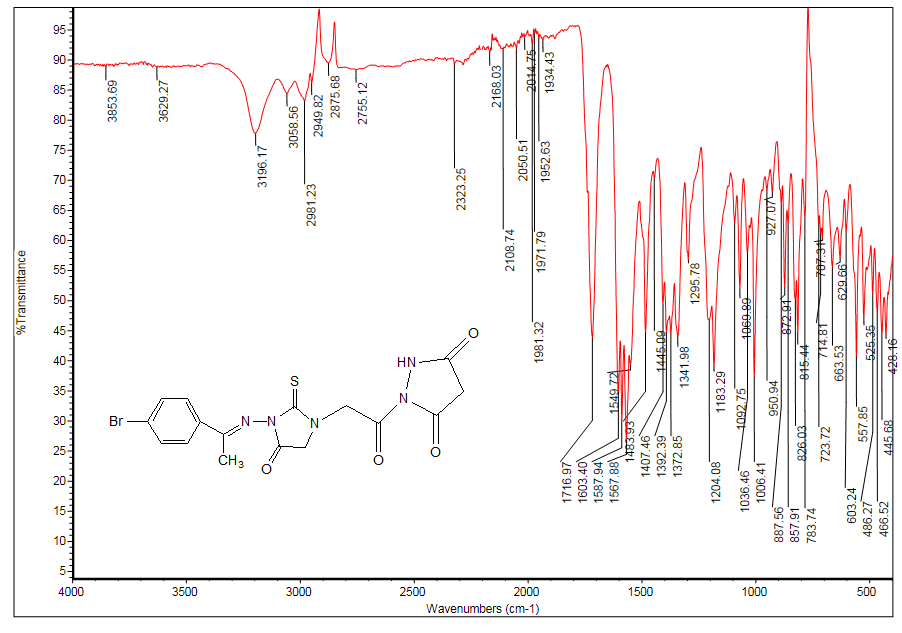


Figure S**18**: IR of compound **7**.


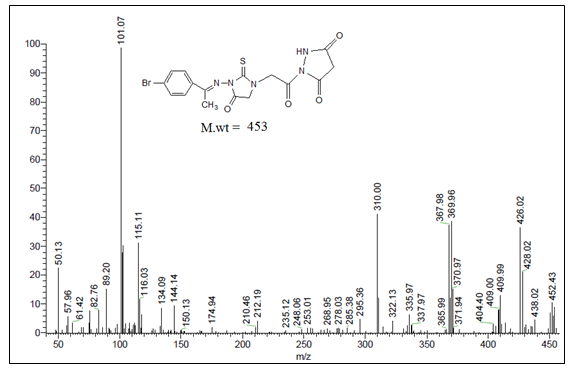


Figure S**19**: Mass spectrum (70 ev) of compound **7**.


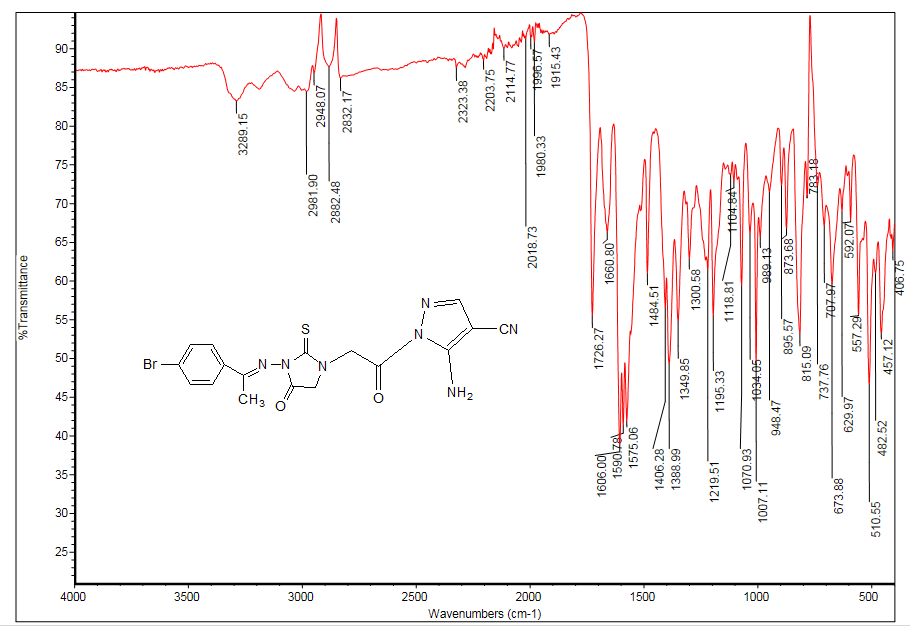


Figure S**20**: IR of compound **8**


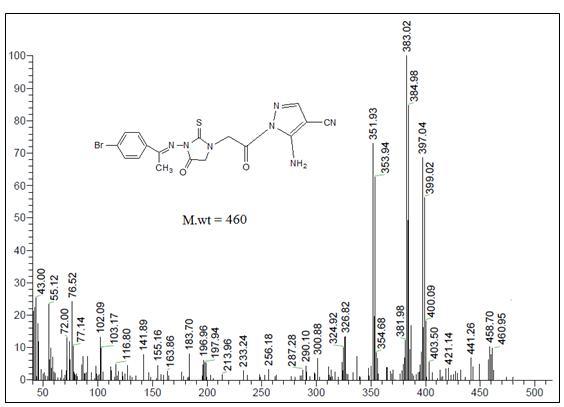


Figure S**21**: Mass spectrum (70 ev) of compound **8**.


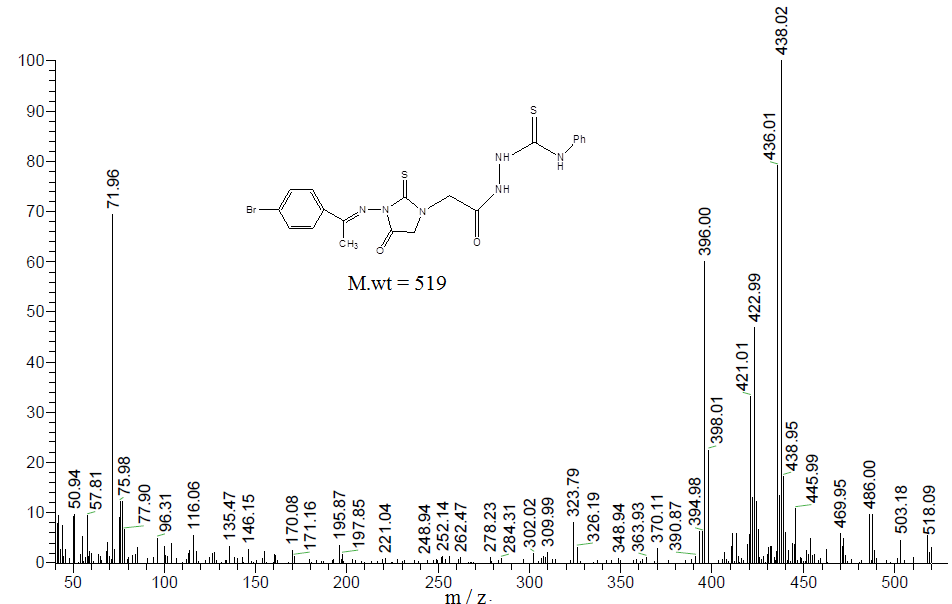


Figure S**22**: Mass spectrum (70 ev) of compound **9**.


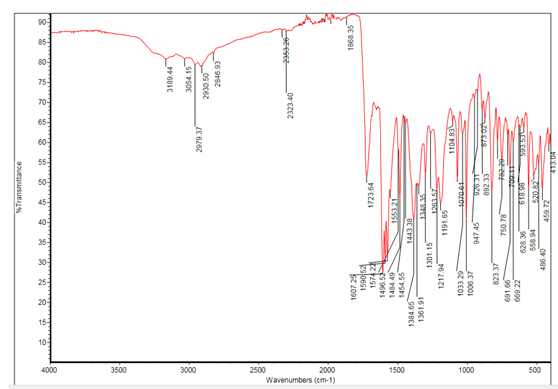


Figure S**23**: IR of compound **10**


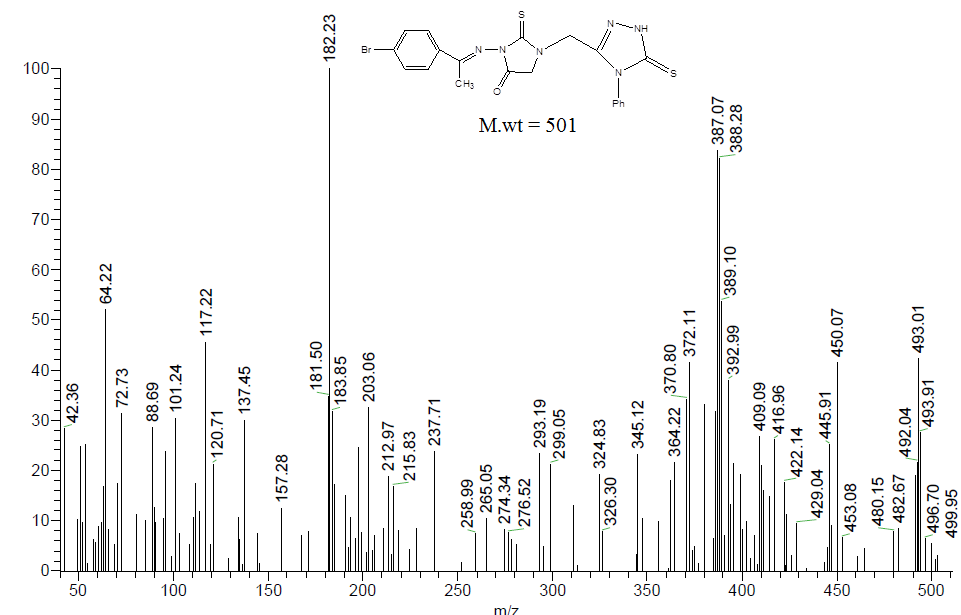


Figure S**24**: Mass spectrum (70 ev) of compound **10**.


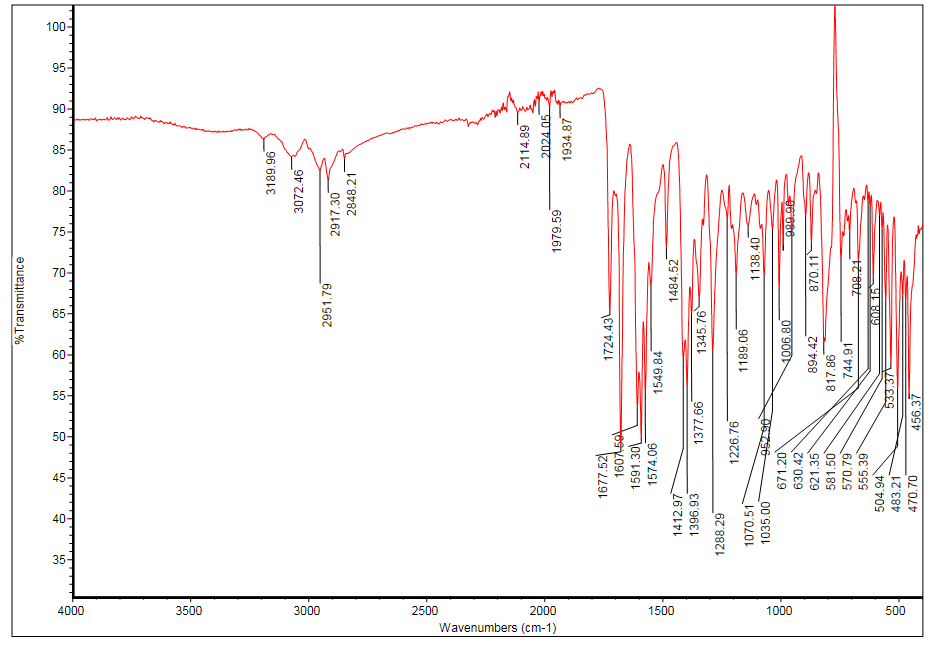


Figure S**25**: IR of compound **11**a.


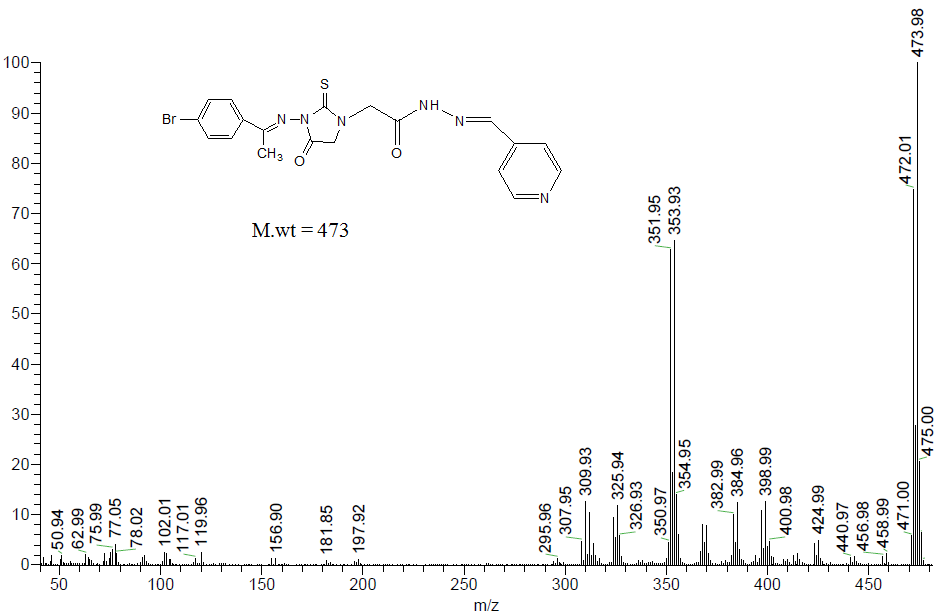


Figure S**26**: Mass spectrum (70 ev) of compound **11**a.


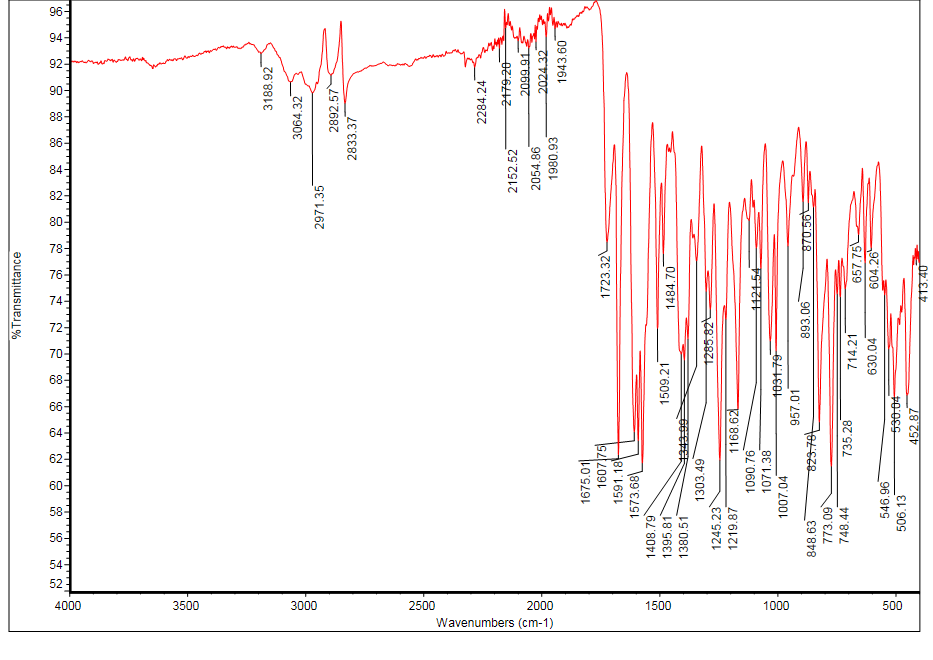


Figure S**27**: IR of compound **11**b.


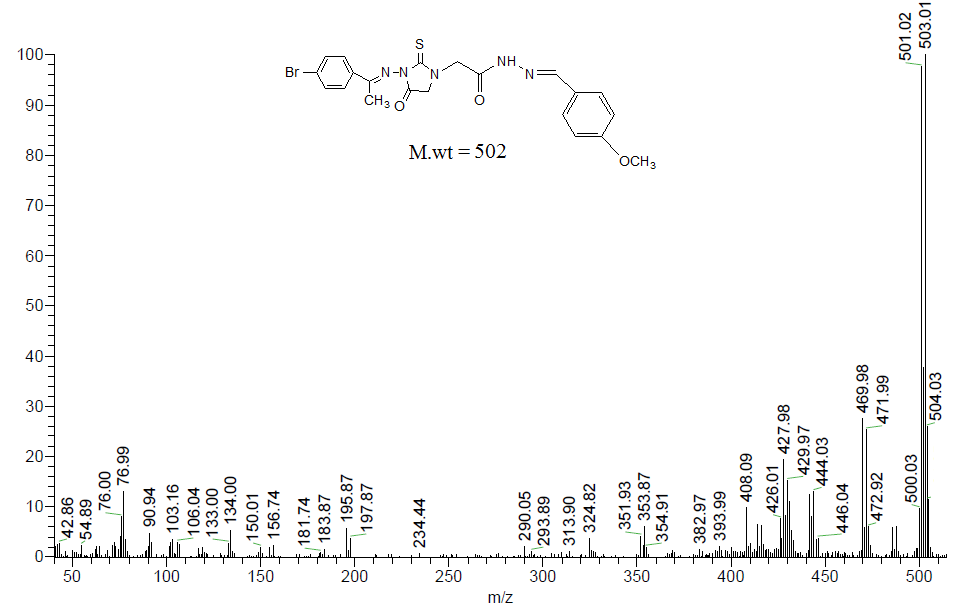


Figure S**28**: Mass spectrum (70 ev) of compound **11**b.

**
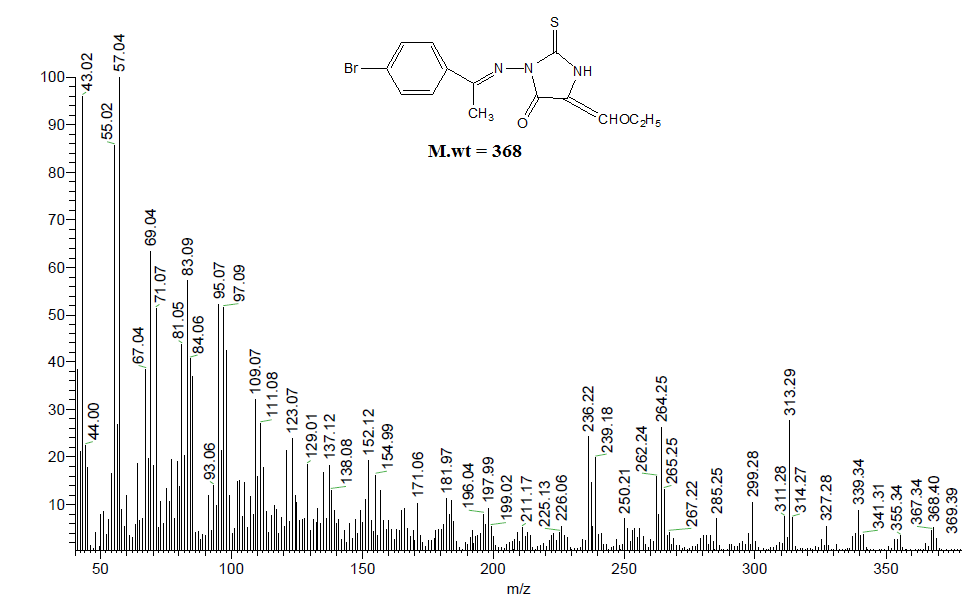
**

Figure S**29**: Mass spectrum (70 ev) of compound **12**


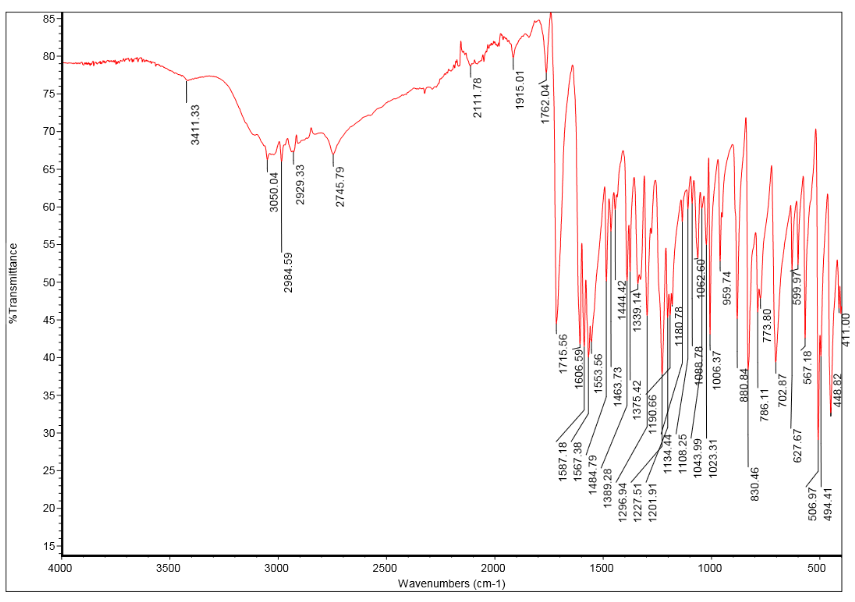


Figure S**30**: IR of compound **13**


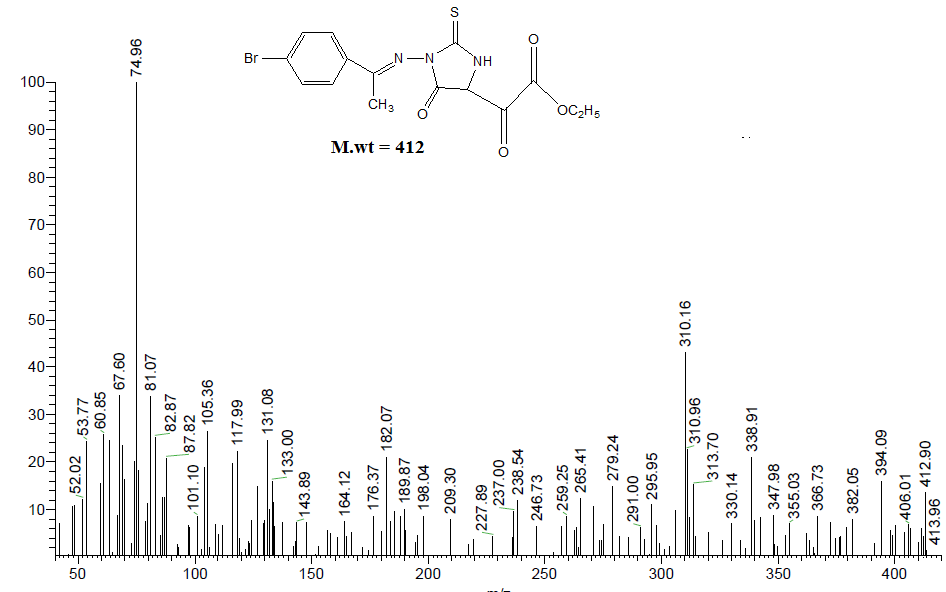


Figure S**31**: Mass spectrum (70 ev) of compound **13**


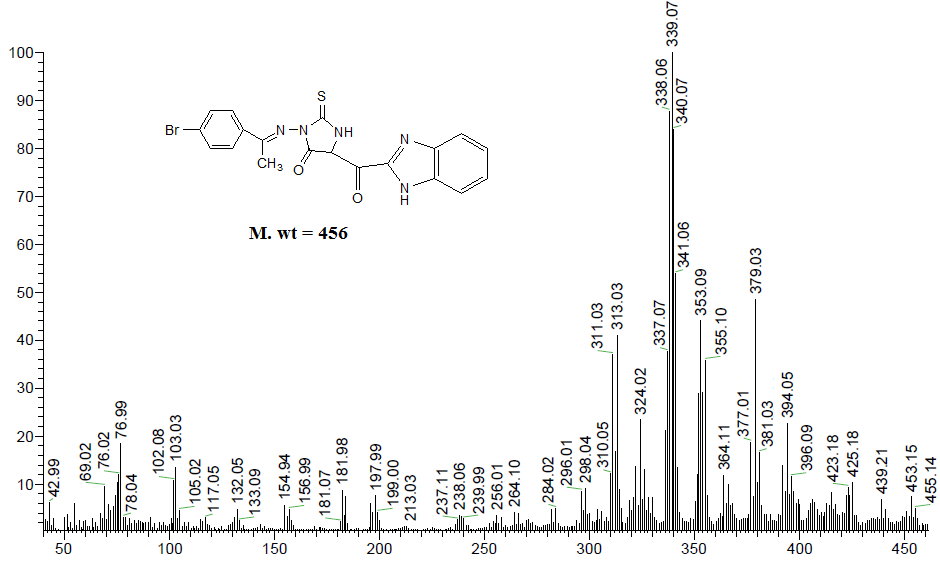


Figure S**32**: Mass spectrum (70 ev) of compound **14**.


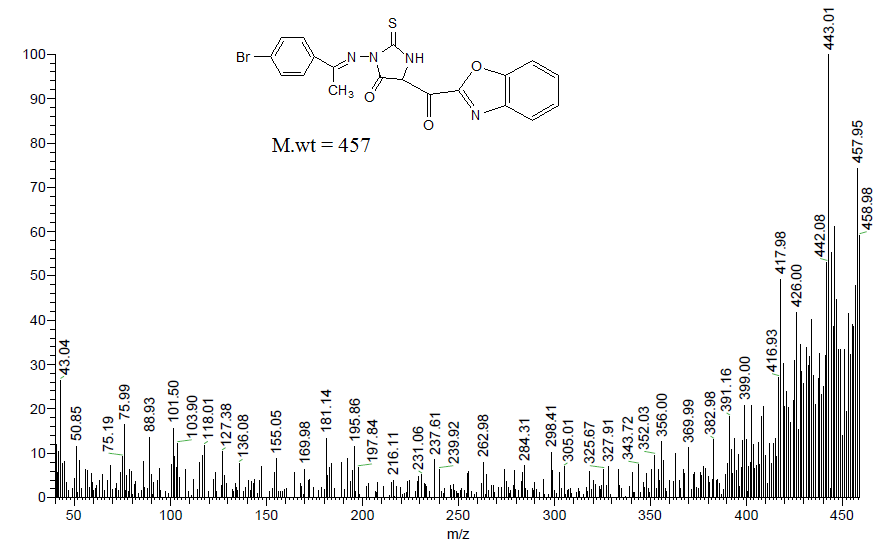


Figure S**33**: Mass spectrum (70 ev) of compound **15**.


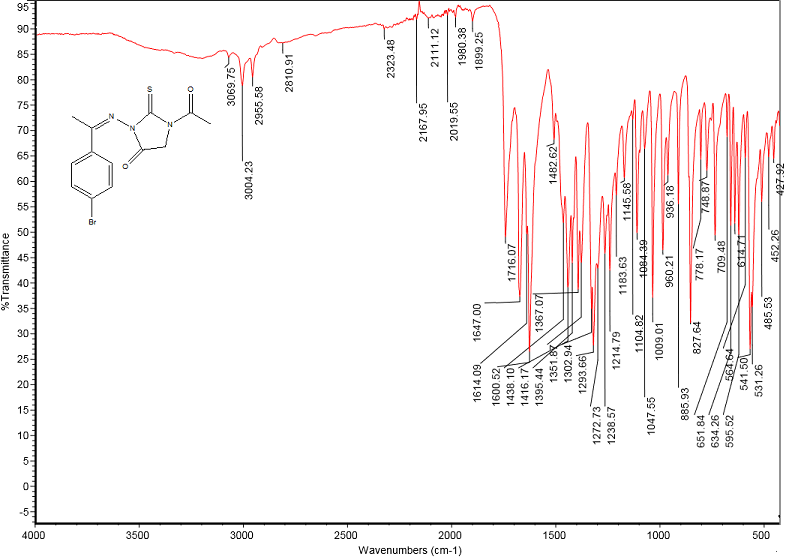
Figure S**34**: IR of compound **16**.


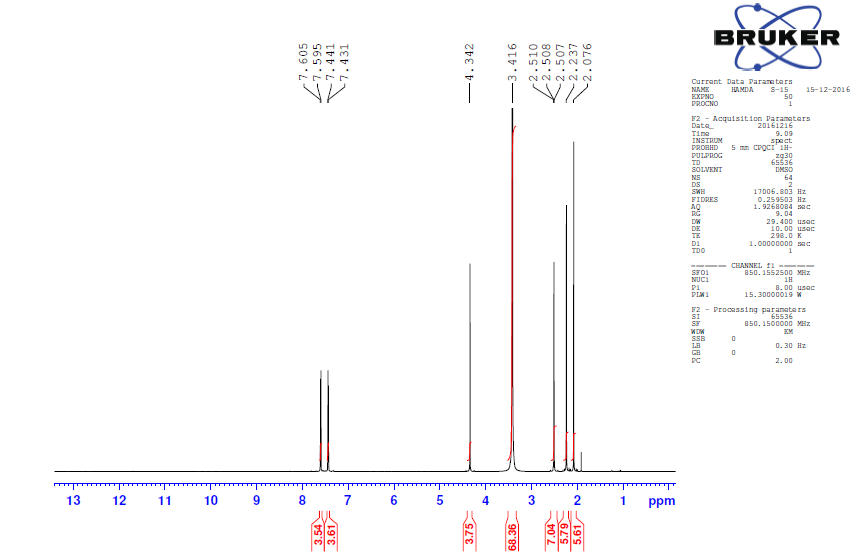


Figure S**35**: ^1^HNMR spectrum of compound **16**


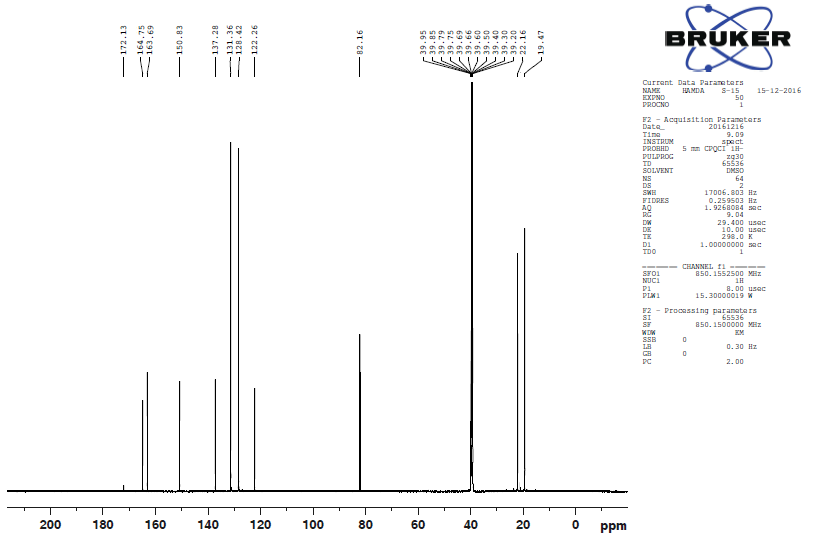


Figure S**36**: ^13^CNMR spectrum of compound **16**.


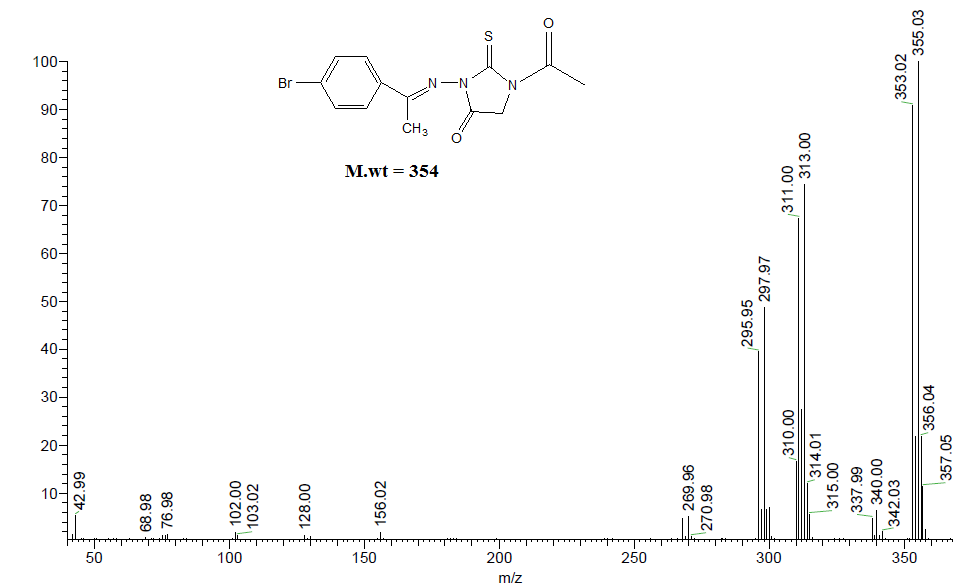


Figure S**37**: Mass spectrum (70 ev) of compound **16**.


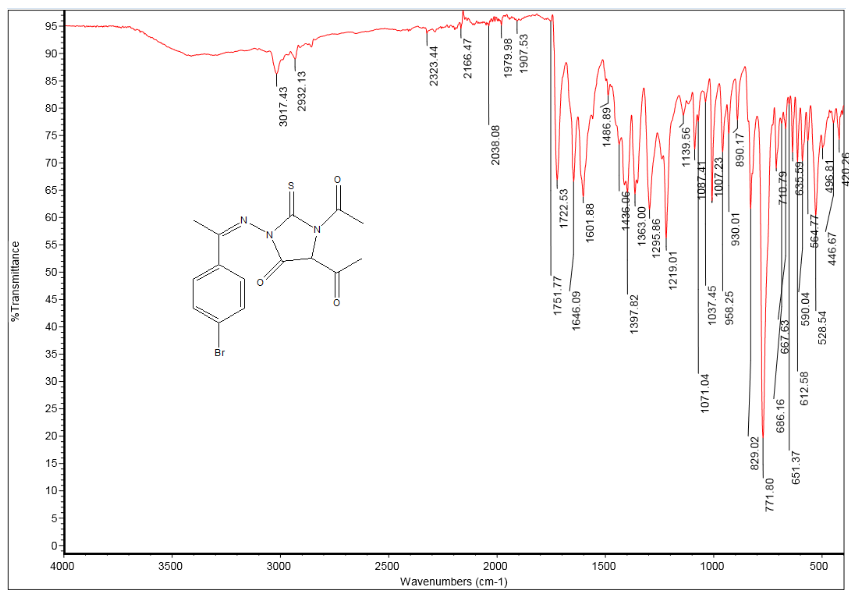


Figure S**38**: IR of compound **17**


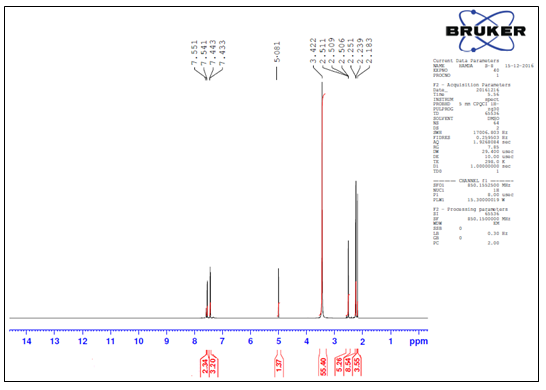


Figure S**39**: ^1^HNMR spectrum of compound **17**.


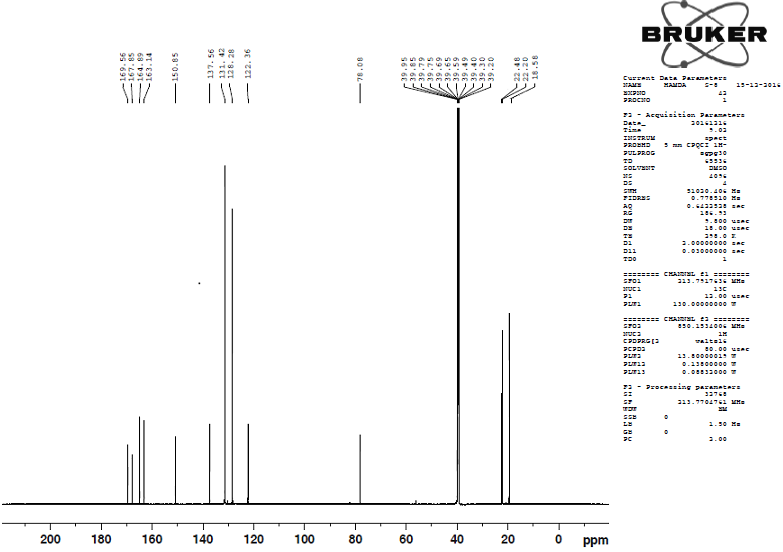


Figure S**40**: ^13^CNMR spectrum of compound **17**.


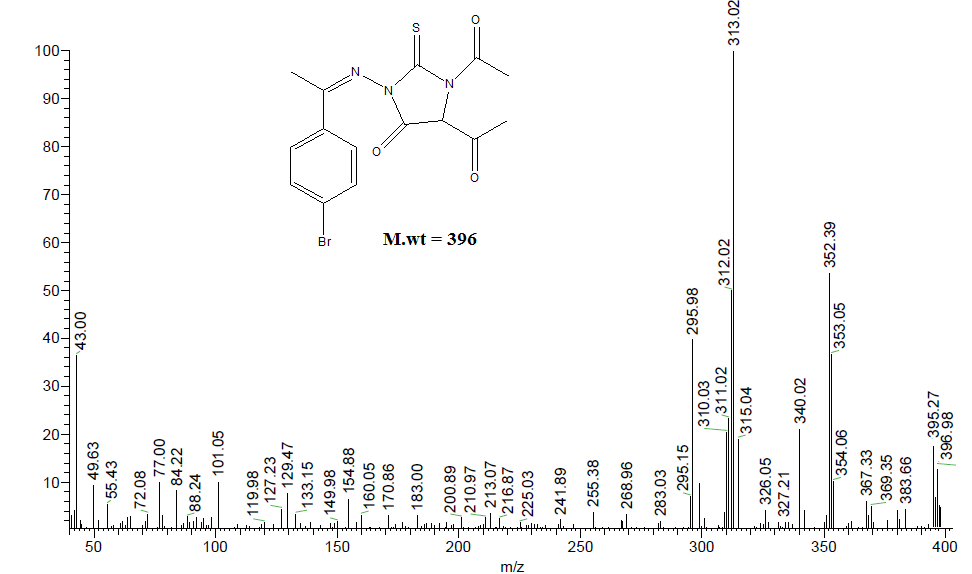


Figure S**41**: Mass spectrum (70 ev) of compound 17**.**


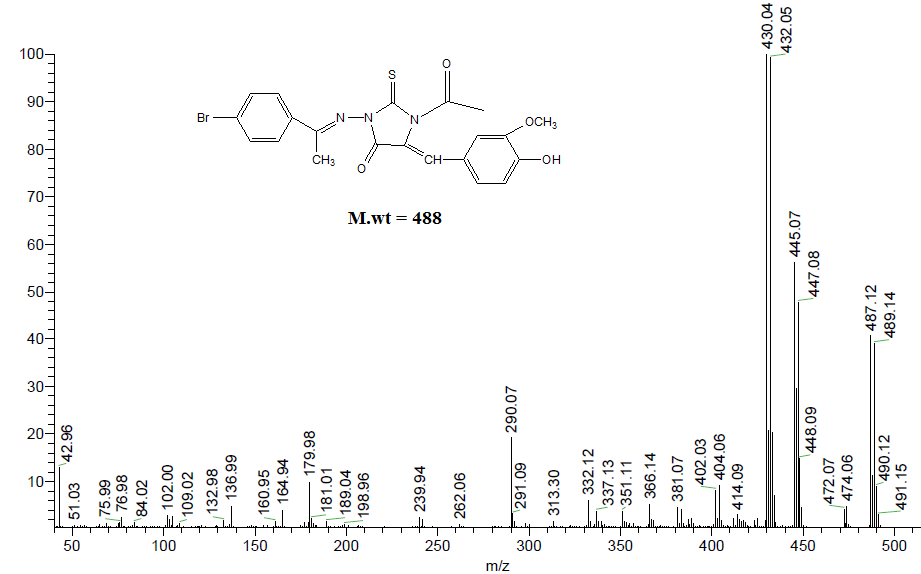


Figure S**42**: Mass spectrum (70 ev) of compound **18**


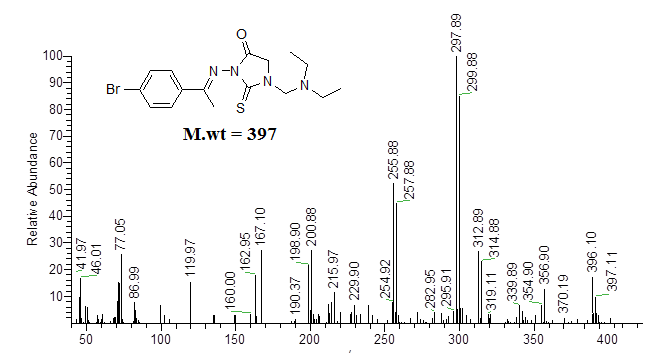


Figure S**43**: Mass spectrum (70 ev) of compound **19**.


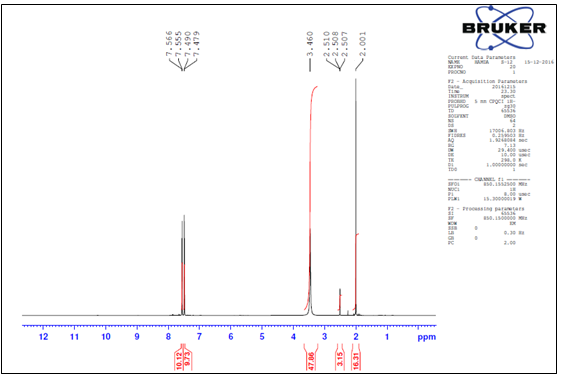


Figure S**44**: ^1^HNMR spectrum of compound **20**.


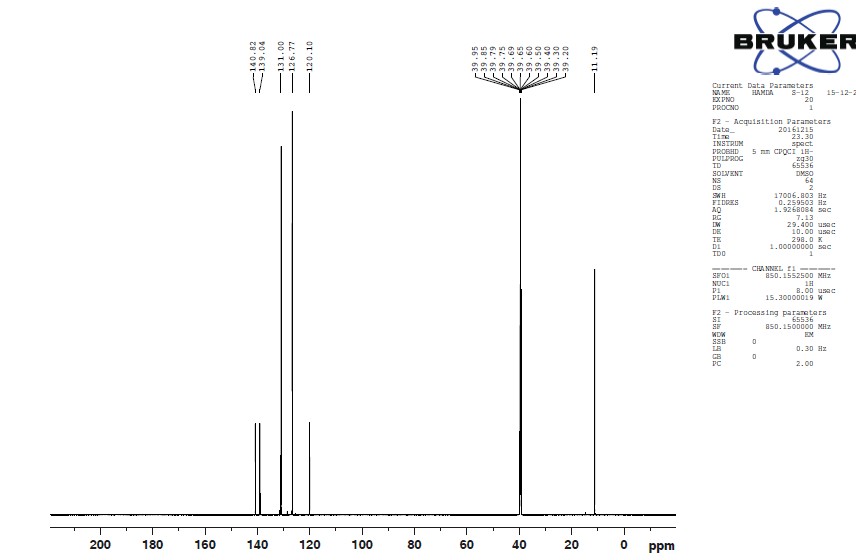


Figure S**45**: ^13^CNMR spectrum of compound **20**.


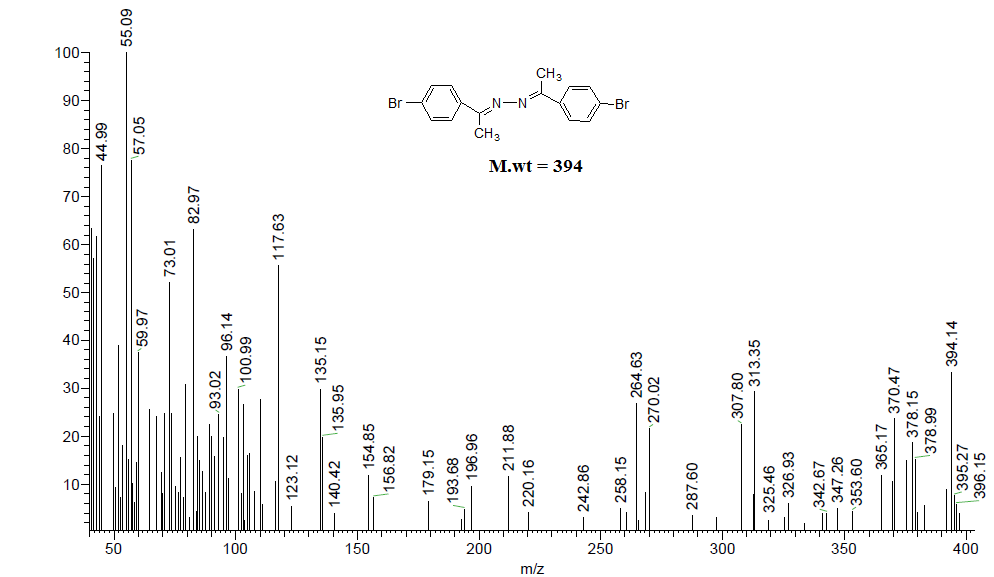


Figure S**46**: Mass spectrum (70 ev) of compound **20**.

Figure S**47**: The drug toxicity curve of compounds **1**, **2**, **3**, **4**, **5**, **6**, **7** and Doxorubicin against hepatocellular carcinoma cell line HePG2.

Figure S**48**: The drug toxicity curve of compounds **8**, **9**, **10**, **11**a, **11**b, **12**, **13** and Doxorubicin against hepatocellular carcinoma cell line HePG2.

Figure **S49**: The drug toxicity curve of compounds **14**, **15**, **16**, **17**, **18**, **19**, **20** and Doxorubicin against hepatocellular carcinoma cell line HePG2.

Figure S**50**: The drug toxicity curve of compounds **1**, **2**, **3**, **4**, **5**, **6**, **7** and Doxorubicin against breast carcinoma cell line MCF-7.

Figure S**51**: The drug toxicity curve of compounds **8**, **9**, **10**, **11**a, **11**b, **12**, **13** and Doxorubicin against breast carcinoma cell line MCF-7.

Figure S**52**: The drug toxicity curve of compounds **14**, **15**, **16**, **17**, **18**, **19**, **20** and Doxorubicin against breast carcinoma cell line MCF-7.
